# Supplementary material for: Scalable cryopreservation of infectious Cryptosporidium hominis oocysts by vitrification
Source: PLoS Pathog. 2023 Jun 8;19(6):e1011425. doi: 10.1371/journal.ppat.1011425 (PMC10284403; doi:10.1371/journal.ppat.1011425)
Supplement: S1 Table — (PDF) [file ppat.1011425.s001.pdf]

Supplementary Table S1. Summary of *C. hominis* infectivity in gnotobiotic piglet after cryopreservation using microcapillary method. Patent infection was established in 100% of piglets inoculated with cryopreserved oocysts with the onset of fecal oocyst shedding on the same day or 1-2 days later than control piglets inoculated with fresh oocysts. The onset of patent infection is indicated as a day post inoculation (dpi) for both piglets infected with cryopreserved and fresh oocysts. *Infectious dose of fresh control oocysts is indicated if different from cryopreserved oocysts dose.

Piglet	Infectious dose	Time in cryopreserved state	Onset of shedding - cryopreserved inoculum (dpi)	Onset of shedding- fresh control inoculum (dpi)
1	10^6	10 min	4	4
2	10^6	2 months	5	4
3	10^5	12 months	4	4
4	10^5	16 months	4	4
5	10^4	16 months	5	4 (10^5)*
6	10^3	16 months	5	4 (10^5)*
7	10^5	40 months	5	3
8	10^5	40 months	5	3
