# Supplementary material for: Scalable cryopreservation of infectious Cryptosporidium hominis oocysts by vitrification
Source: PLoS Pathog. 2023 Jun 8;19(6):e1011425. doi: 10.1371/journal.ppat.1011425 (PMC10284403; doi:10.1371/journal.ppat.1011425)
Supplement: S1 Fig — (PDF) [file ppat.1011425.s002.pdf]

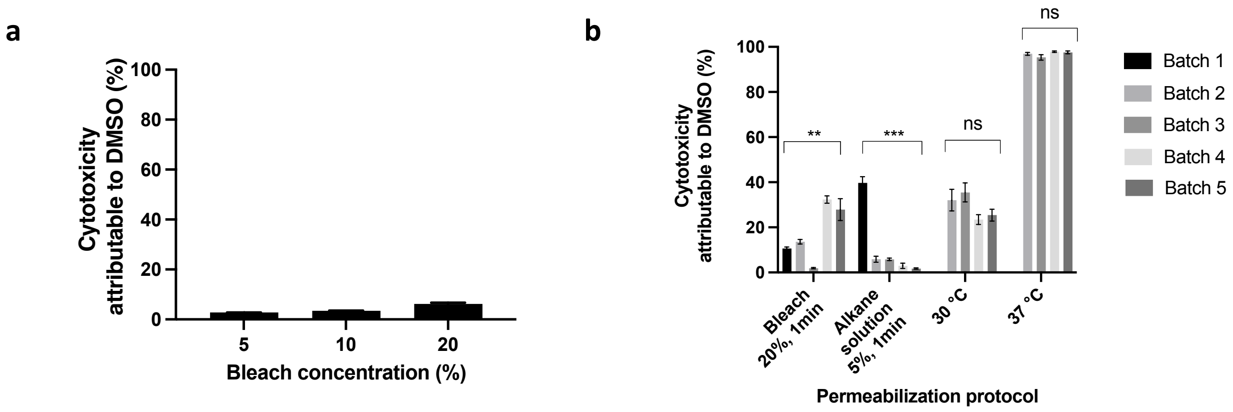

Supplementary Figure S1. Chemical permeabilization of *C. hominis* results in variable cryoprotective agent uptake. a) Bleaching does not permeabilize 2-week-old oocysts to CPA uptake. Oocysts exposed to 0.5 M trehalose/30% DMSO for 30 min following treatment with bleach (5-20%) remain viable as measured by PI exclusion using flow cytometry. Values indicate mean and bars indicate standard error (n = 2). **b)** In contrast to thermal permeabilization, chemical permeabilization leads to variable CPA uptake across five batches of oocysts matched by age at 6-7 weeks. Oocysts were exposed to 0.5 M trehalose/50% DMSO for 30 min either after chemical treatment with bleach or alkane solution, or during thermal treatment at 30 °C or 37 °C. DMSO-induced mortality measured by PI inclusion was recorded by flow cytometry. Values indicate mean and bars indicate standard error ($n \geq 3$). Differential response was observed in oocyst treated with bleach (Brown-Forsythe ANOVA; $^{**}p = 0.004$, F = 26.41, df = 4, Shapiro-Wilk normality test; $p > 0.11$, Brown-Forsythe; $p = 0.0001$) and alkane solution (Kruskal-Wallis; $^{***}p = 0.0006$; data normality requirement for parametric test not satisfied; Brown–Forsythe homoscedasticity test; $p = 0.22$). Thermally treated oocysts responded invariably to DMSO uptake both at 30 °C (One-way ANOVA; $p = 0.18$, F = 1.83, df = 3, Shapiro-Wilk normality test; $p > 0.27$, Brown–Forsythe homoscedasticity test; $p = 0.26$) and 37 °C (One-way ANOVA; $p = 0.13$, F = 2.33, df = 3, Shapiro-Wilk normality test; $p > 0.52$, Brown–Forsythe homoscedasticity test; $p = 0.5$). Ns= non-significant.
