# Supplementary material for: Scalable cryopreservation of infectious Cryptosporidium hominis oocysts by vitrification
Source: PLoS Pathog. 2023 Jun 8;19(6):e1011425. doi: 10.1371/journal.ppat.1011425 (PMC10284403; doi:10.1371/journal.ppat.1011425)
Supplement: S2 Fig — (PDF) [file ppat.1011425.s003.pdf]

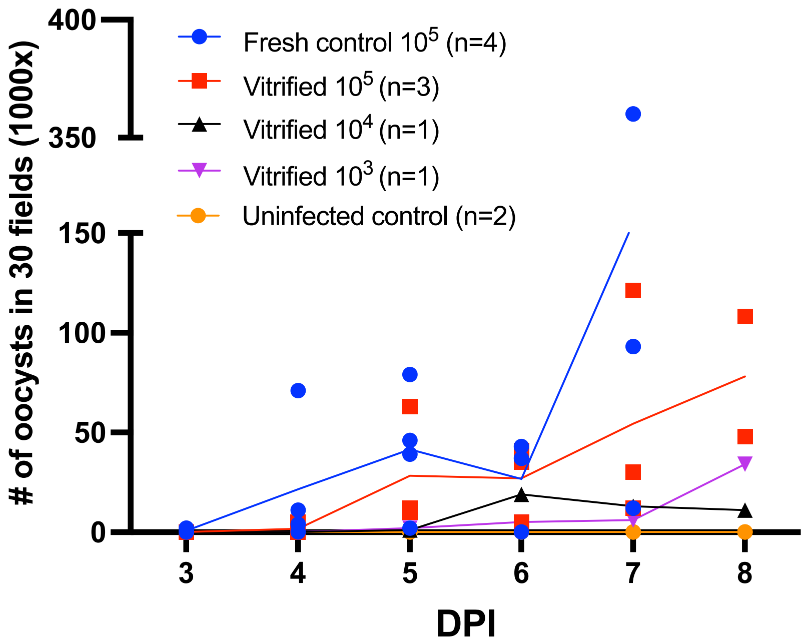

Supplementary Figure S2. ***C. hominis*** **oocysts vitrified in microcapillaries are infectious to gnotobiotic piglets (untransformed individual data plot).** *C. hominis* oocysts are infectious to gnotobiotic piglets after 16-40 months of cryogenic storage. To determine the minimum infectious dose, piglets were inoculated orally with either 10^5 (n = 3), 10^4 (n = 1) or 10^3 thawed PI^- oocysts (n = 1) in the presence of controls infected with 10^5 fresh oocysts (n = 4) and uninfected controls (n = 2). Aside from two inocula dosed at 10^5 oocysts, which were recovered after 40 months of cryogenic storage, the remainder of inocula were stored for 16 months. Fecal shedding of oocysts was determined daily by microscopic enumeration in 30 fields of acid-fast stained fecal smears examined at 1000x magnification. Data points indicate absolute oocyst counts and the line represents the mean. Figure 3c reports the mean and standard error of pooled log transformed data.
