# Supplementary material for: Scalable cryopreservation of infectious Cryptosporidium hominis oocysts by vitrification
Source: PLoS Pathog. 2023 Jun 8;19(6):e1011425. doi: 10.1371/journal.ppat.1011425 (PMC10284403; doi:10.1371/journal.ppat.1011425)
Supplement: S3 Fig — (PDF) [file ppat.1011425.s004.pdf]

```
                                10        20        30        40        50        60        70        80        90       100
                        ....|....|....|....|....|....|....|....|....|....|....|....|....|....|....|....|....|....|....|....|
C. parvum_IOWA          CACATTAAATGTGACATATCATTCAAGTTTCTGACCTATCAGCTTTAGACGGTAGGGTATTGGCCTACCGTGGCAATGACGGGTAACGGGGAATTAGGGT
C. hominis Chro.rrn022  ....A.T.............................................................................................
C. hominis before cryo  ....A.T.............................................................................................
C. hominis cryo         ....A.T.............................................................................................

                               110       120       130       140       150       160       170       180       190       200
                        ....|....|....|....|....|....|....|....|....|....|....|....|....|....|....|....|....|....|....|....|
C. parvum IOWA          TCGATTCCGGAGAGGGAGCCTGAGAAACGGCTACCACATCTAAGGAAGGCAGCAGGCGCGCAAATTACCCAATCCTAATACAGGGAGGTAGTGACAAGAA
C. hominis Chro.rrn022  ....................................................................................................
C. hominis before cryo  ....................................................................................................
C. hominis cryo         ....................................................................................................

                               210       220       230       240       250       260       270       280       290       300
                        ....|....|....|....|....|....|....|....|....|....|....|....|....|....|....|....|....|....|....|....|
C. parvum IOWA          ATAACAATACAGGACTTTTTGGTTTTGTAATTGGAATGAGTTAAGTATAAACCCCTTTACAAGTATCAATTGGAGGGCAAGTCTGGTGCCAGCAGCCGCG
C. hominis Chro.rrn022  ....................................................................................................
C. hominis before cryo  ....................................................................................................
C. hominis cryo         ....................................................................................................

                               310       320       330       340       350       360       370       380       390       400
                        ....|....|....|....|....|....|....|....|....|....|....|....|....|....|....|....|....|....|....|....|
C. parvum IOWA          GTAATTCCAGCTCCAATAGCGTATATTAAAGTTGTTGCAGTTAAAAAGCTCGTAGTTGGATTTCTGTTAATAATTTATATAAAATATTTTGATGAATATT
C. hominis Chro.rrn022  ....................................................................................................
C. hominis before cryo  ....................................................................................................
C. hominis cryo         ....................................................................................................

                               410       420       430       440       450       460       470       480       490       500
                        ....|....|....|....|....|....|....|....|....|....|....|....|....|....|....|....|....|....|....|....|
C. parvum IOWA          TATATAATATTAACATAATTCATATTACTATATATTT---TAGTATATGAAATTTTACTTTGAGAAAATTAGAGTGCTTAAAGCAGGCATATGCCTTGAA
C. hominis Chro.rrn022  ...............................T.T...TTT............................................................
C. hominis before cryo  ...............................T.T...TTT............................................................
C. hominis cryo         ...............................T.T...TTT............................................................

                               510       520       530       540       550       560       570       580       590       600
                        ....|....|....|....|....|....|....|....|....|....|....|....|....|....|....|....|....|....|....|....|
C. parvum IOWA          TACTCCAGCATGGAATAATATTAAAGATTTTTATCTTTCTTATTGGTTCTAAGATAAGAATAATGATTAATAGGGACAGTTTGGGGGCATTTGTATTTAA
C. hominis Chro.rrn022  ......................................T..........................................-..................
C. hominis before cryo  ......................................T..........................................-..................
C. hominis cryo         ......................................T..........................................-..................

                               610       620       630
                        ....|....|....|....|....|....|....|....
C. parvum IOWA          CAGTCAGAGGTGAAATTCTTAGATTTGTTAAAGACAAAC
C. hominis Chro.rrn022  .......................................
C. hominis before cryo  .......................................
C. hominis cryo         .......................................
```

Supplementary Figure S3. Alignment of 18s rRNA gene from *C. hominis* oocysts before and after cryopreservation in microcapillaries. A portion of the 18S ribosomal RNA gene was amplified from *C. hominis* oocysts recovered from piglets after 12 months of cryogenic storage in microcapillaries for comparison with the gene of matched oocysts before cryopreservation and the reference gene of *C. hominis* (AF093491). The corresponding sequence amplified from *C. parvum* (Iowa) oocysts was included as a control. Sequences were aligned with ClustalW software and the alignment displayed with BioEdit 7.2 (1). All sequences from *C. hominis* are identical and differ from that *of C. parvum*.

References:

1. Hall TA. BioEdit: a user-friendly biological sequence alignment editor and analysis program for Widnows 95/98/NT. Nucleic Acids Symposium Series. 1999.
