# Supplementary material for: Scalable cryopreservation of infectious Cryptosporidium hominis oocysts by vitrification
Source: PLoS Pathog. 2023 Jun 8;19(6):e1011425. doi: 10.1371/journal.ppat.1011425 (PMC10284403; doi:10.1371/journal.ppat.1011425)
Supplement: S4 Fig — (PDF) [file ppat.1011425.s005.pdf]

a

b

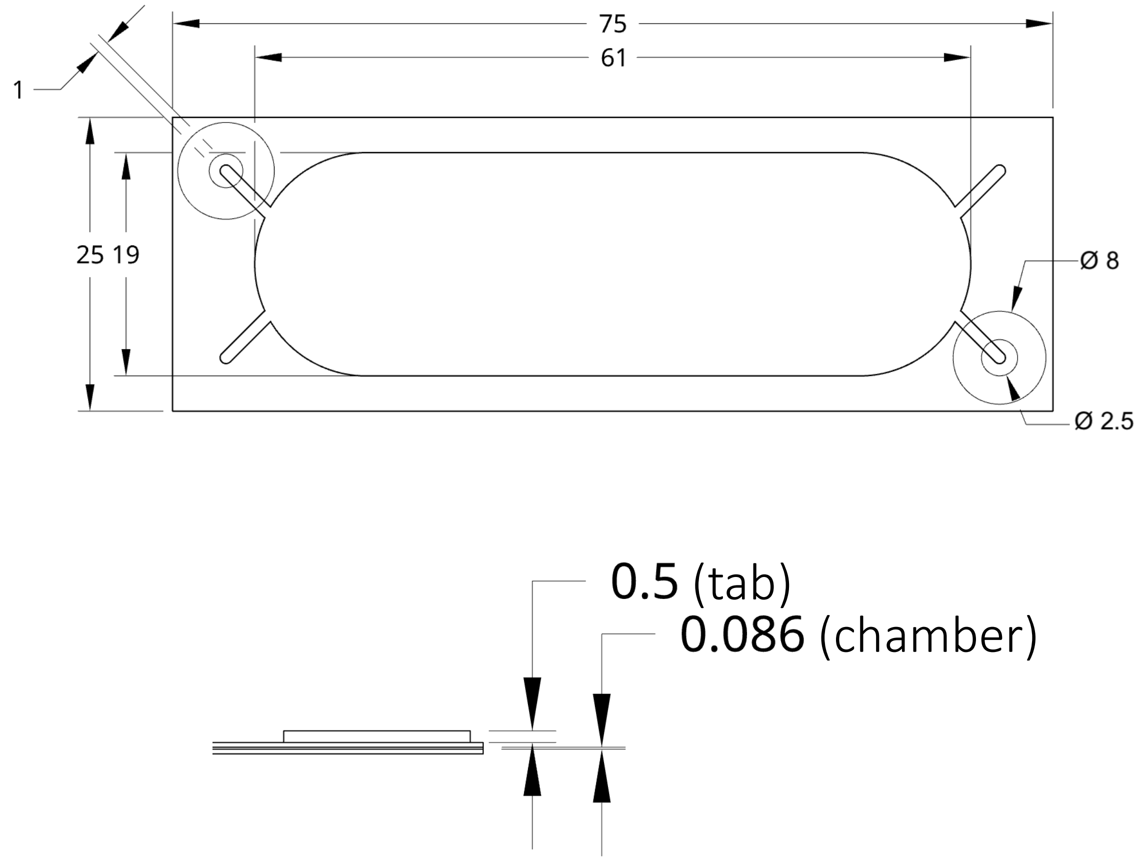

Supplementary Figure S4. High aspect ratio cassette design. Detailed cassette dimensions are provided in mm for **a)** top view and **b)** cross sectional view.
