# Supplementary material for: Scalable cryopreservation of infectious Cryptosporidium hominis oocysts by vitrification
Source: PLoS Pathog. 2023 Jun 8;19(6):e1011425. doi: 10.1371/journal.ppat.1011425 (PMC10284403; doi:10.1371/journal.ppat.1011425)
Supplement: S5 Fig — (PDF) [file ppat.1011425.s006.pdf]

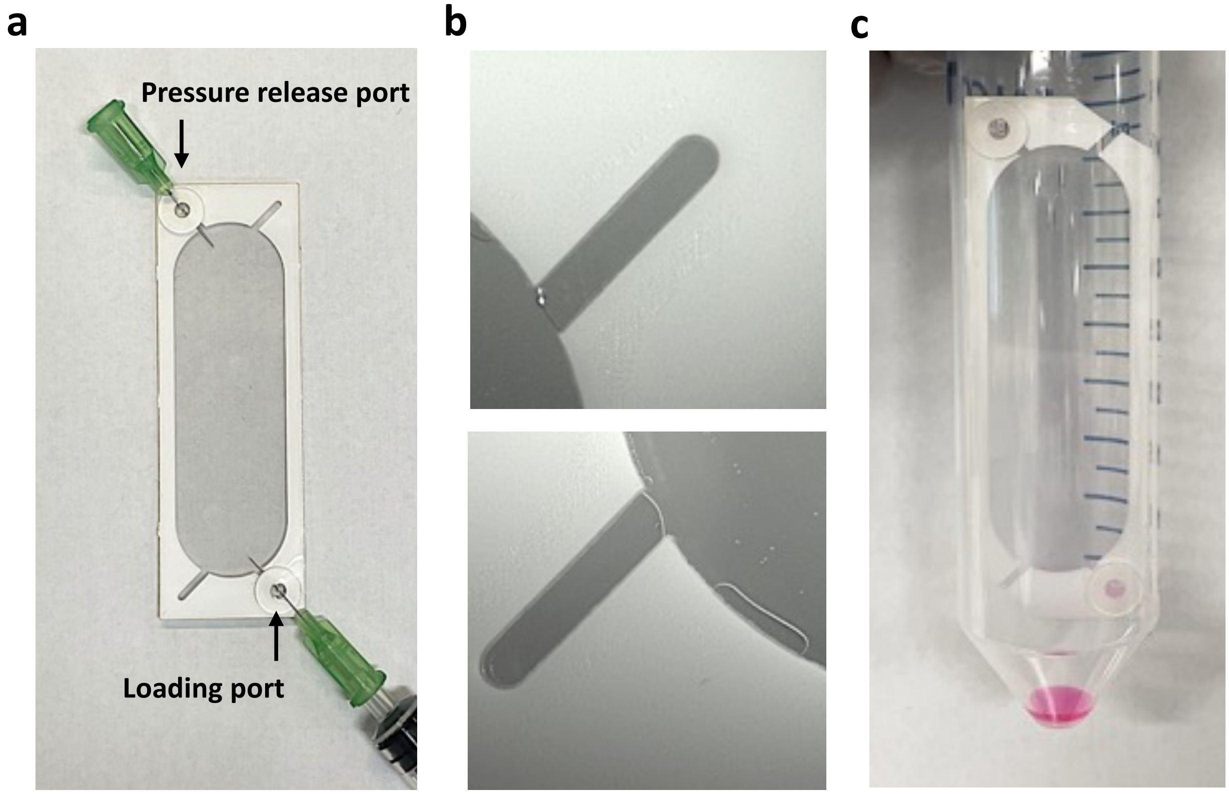

Supplementary Figure S5. Strategy for cassette loading and unloading. a) Sample is loaded by puncturing through a silicone tab at an angle with a blunt 28G needle affixed to a syringe. During loading, the opposite tab is also perforated with a needle to remove displaced air and ensure uniform sample distribution within the device. **b)** The exit ports are designed as dead-end channels to prevent entry of biospecimen and to avoid splashes during cutting **c)** After both exit channels are cut open with scissors, the sample is recovered by centrifugation in a 50 ml conical tube (200 ×g, 1 min). Only 2-3% of the sample volume remains in the cassette after centrifugation. The sample is colored with a pink dye to visualize recovered sample and residual volume remaining in the cassette.
