# Supplementary material for: Scalable cryopreservation of infectious Cryptosporidium hominis oocysts by vitrification
Source: PLoS Pathog. 2023 Jun 8;19(6):e1011425. doi: 10.1371/journal.ppat.1011425 (PMC10284403; doi:10.1371/journal.ppat.1011425)
Supplement: S6 Fig — (PDF) [file ppat.1011425.s007.pdf]

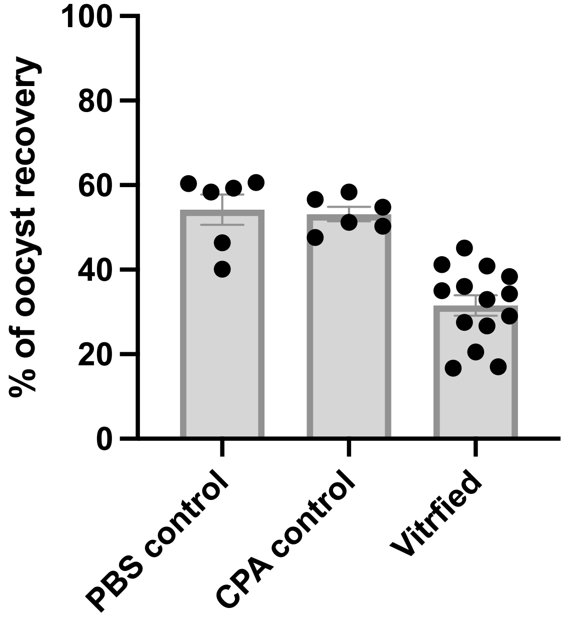

Supplementary Figure S6. Recovery of oocyst sample from cassette. The percent of full oocysts recovered from the cassette was determined. Cassettes were loaded with 10^6 *C. hominis* oocysts suspended either in PBS or 0.5 M trehalose/50% DMSO after 2 min exposure at 37 °C. CPA-loaded oocysts were either cryopreserved in liquid nitrogen (vitrified) or immediately unloaded without cryopreservation to serve as a control (CPA control). Unloaded oocysts were incubated in excess PBS for 30 min followed by centrifugation. Procedural steps at which oocyst number is reduced are cassette unloading and centrifugation. Additional loss to lysis is introduced by cryopreservation. Error bars indicate standard error ($n \geq 6$).
