# Supplementary material for: Scalable cryopreservation of infectious Cryptosporidium hominis oocysts by vitrification
Source: PLoS Pathog. 2023 Jun 8;19(6):e1011425. doi: 10.1371/journal.ppat.1011425 (PMC10284403; doi:10.1371/journal.ppat.1011425)
Supplement: S7 Fig — (PDF) [file ppat.1011425.s008.pdf]

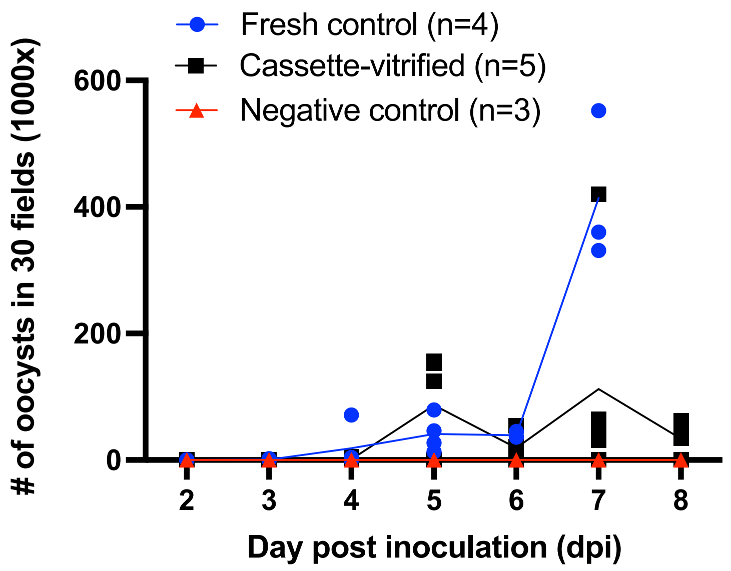

Supplementary Figure S7. ***C. hominis*** **oocysts aged < 10 weeks vitrified in cassettes are infectious to gnotobiotic piglets (untransformed individual data plot).** Oocysts were cryopreserved in cassettes using the 2-min protocol of 0.5 M trehalose/50% DMSO exposure at 37 °C. Gnotobiotic piglets were inoculated orally with 500,000 thawed PI^- oocysts (n = 5) in the presence of controls infected with 500,000 fresh matched oocysts (n = 4) and negative controls (n = 3). Fecal shedding of oocysts was determined daily by microscopic enumeration in 30 fields of acid-fast stained fecal smears examined under 1000x magnification. Two out of three piglets produced a patent infection 1-3 days later than controls infected with fresh parasite. Data points indicate absolute oocyst counts and the line represents the mean. Figure 6a reports the mean and standard error of pooled log transformed data.
