# Supplementary material for: Scalable cryopreservation of infectious Cryptosporidium hominis oocysts by vitrification
Source: PLoS Pathog. 2023 Jun 8;19(6):e1011425. doi: 10.1371/journal.ppat.1011425 (PMC10284403; doi:10.1371/journal.ppat.1011425)
Supplement: S8 Fig — (PDF) [file ppat.1011425.s009.pdf]

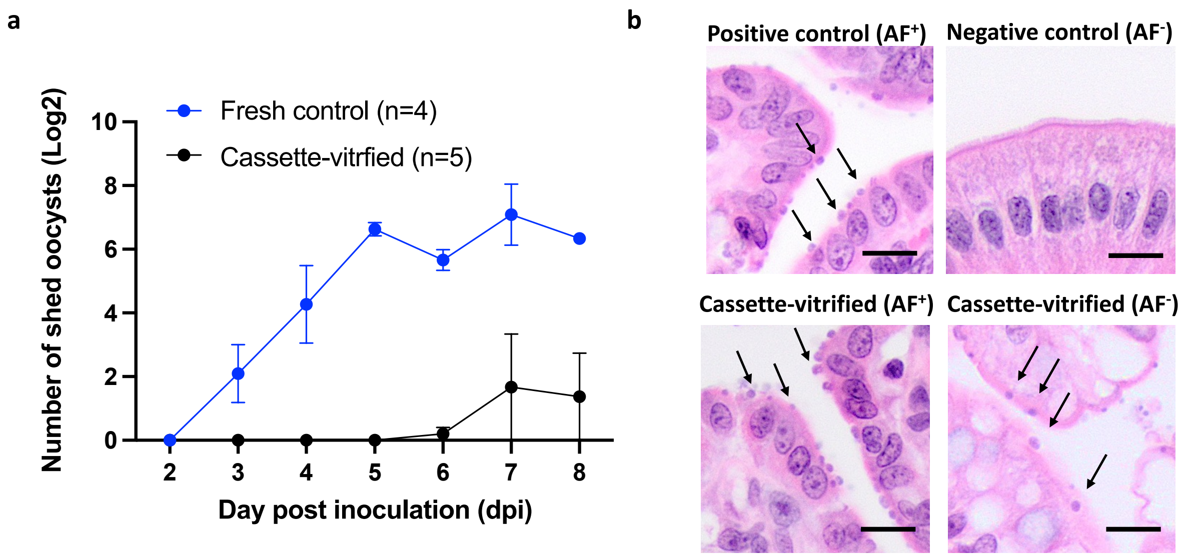

Supplementary Figure S8. Reduction in infectivity of *C. hominis* aged > 12 weeks cryopreserved in cassettes using 37 °C permeabilization protocol. *C. hominis* oocysts aged 11-14 weeks originating from two different batches were cryopreserved in cassettes using 2 min protocol of 0.5 M trehalose/50% DMSO exposure at 37 °C. Gnotobiotic piglets received orally either 500,000 thawed PI^- oocysts (n=5) or 500,000 fresh matched control oocysts (n=4). **a)** Fecal shedding of oocysts was determined daily by microscopic enumeration in 30 fields of acid-fast stained fecal smears examined under 1000x magnification. Values indicate mean of log transformed oocyst counts and bars indicate standard error. Onset of patent infection was observed in one animal marking infectivity rate at 20% (1/5). **b)** Hematoxylin and eosin-stained sections from piglets show intracellular parasite stages located at the apex of enterocytes in both piglets tested negative and positive by acid fast (AF) staining. Scale indicates 20 µm. Although onset of patent infection was observed in 20% of animals, 80% animals became infected as evident on histological examination. Observed delay in the onset of fecal oocyst shedding and lower infectivity rate indicate that cryopreservation of > 11-weeks-old oocysts yields less viable parasite.
