# Supplementary material for: Scalable cryopreservation of infectious Cryptosporidium hominis oocysts by vitrification
Source: PLoS Pathog. 2023 Jun 8;19(6):e1011425. doi: 10.1371/journal.ppat.1011425 (PMC10284403; doi:10.1371/journal.ppat.1011425)
Supplement: S9 Fig — (PDF) [file ppat.1011425.s010.pdf]

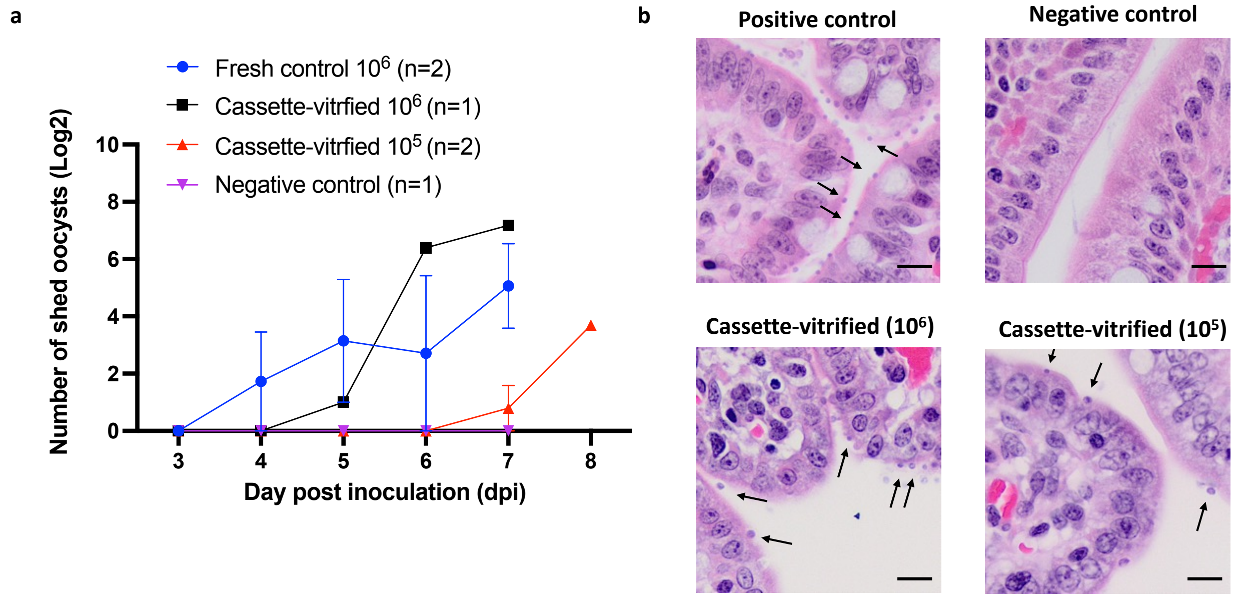

Supplementary Figure S9. Infectivity of *C. hominis* cryopreserved in cassettes using 30 °C permeabilization protocol. Thirteen-week-old oocyst originating from a single batch were cryopreserved in cassettes using 2 min protocol of 0.5 M trehalose/50% DMSO exposure at 30 °C. Gnotobiotic piglets were inoculated orally with either 1,000,000 (n = 1) or 100,000 (n = 2) thawed PI$^-$ oocysts in presence of controls infected with 1,000,000 fresh matched oocysts (n = 2) and an uninfected control (n = 1). **a)** Fecal shedding of oocysts was determined daily by microscopic enumeration in 30 fields of acid-fast stained fecal smears examined under 1000x magnification. Values indicate mean of log transformed oocyst counts and bars indicate standard error. The piglet infected with a matching dose produced a patent infection 1 day later than controls infected with fresh parasite. For piglets infected with 10-fold lower dose, onset of fecal oocyst shedding was delayed by 3 days. **b)** Micrographs of hematoxylin and eosin-stained ileal sections from piglets inoculated with oocysts, either fresh or cryopreserved, and from an uninfected control are shown. Arrows indicate intracellular parasite stages located at the apex of enterocytes. Scale indicates 20 µm.
