# Supplementary material for: Scalable cryopreservation of infectious Cryptosporidium hominis oocysts by vitrification
Source: PLoS Pathog. 2023 Jun 8;19(6):e1011425. doi: 10.1371/journal.ppat.1011425 (PMC10284403; doi:10.1371/journal.ppat.1011425)
Supplement: S10 Fig — (PDF) [file ppat.1011425.s011.pdf]

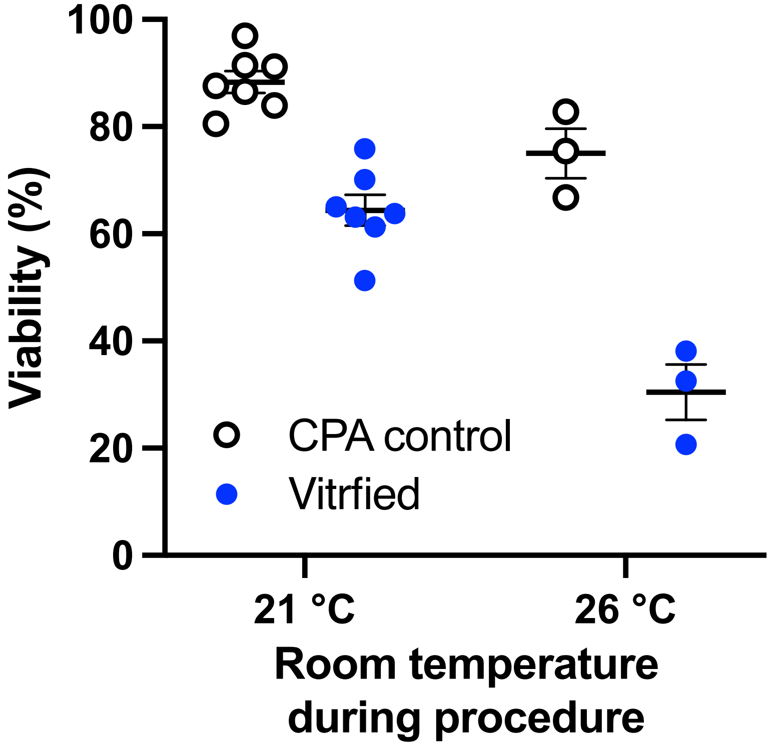

Supplementary Figure S10. Cryopreservation protocol is sensitive to variation in room temperature. *C. hominis* oocysts were cryopreserved in cassettes using the 2 min protocol of 0.5 M trehalose/50% DMSO exposure at 37 °C. A marked decrease in viability after thawing was observed in trials performed at surrounding temperature of 26 °C. Data points indicate individual values; lines indicate the mean and bars indicate the standard error. Permeation of 50% DMSO into oocysts likely continues at 26 °C during cassette loading and unloading, resulting in lower viability of recovered parasite. Cryopreserved oocysts handled at ambient temperature established infection in 100% of piglets ($n = 3$), while those handled at 26 °C infected only 33% of piglets ($n = 3$), as determined by microscopic observation of ileal sections.
