# Supplementary material for: Scalable cryopreservation of infectious Cryptosporidium hominis oocysts by vitrification
Source: PLoS Pathog. 2023 Jun 8;19(6):e1011425. doi: 10.1371/journal.ppat.1011425 (PMC10284403; doi:10.1371/journal.ppat.1011425)
Supplement: S11 Fig — (PDF) [file ppat.1011425.s012.pdf]

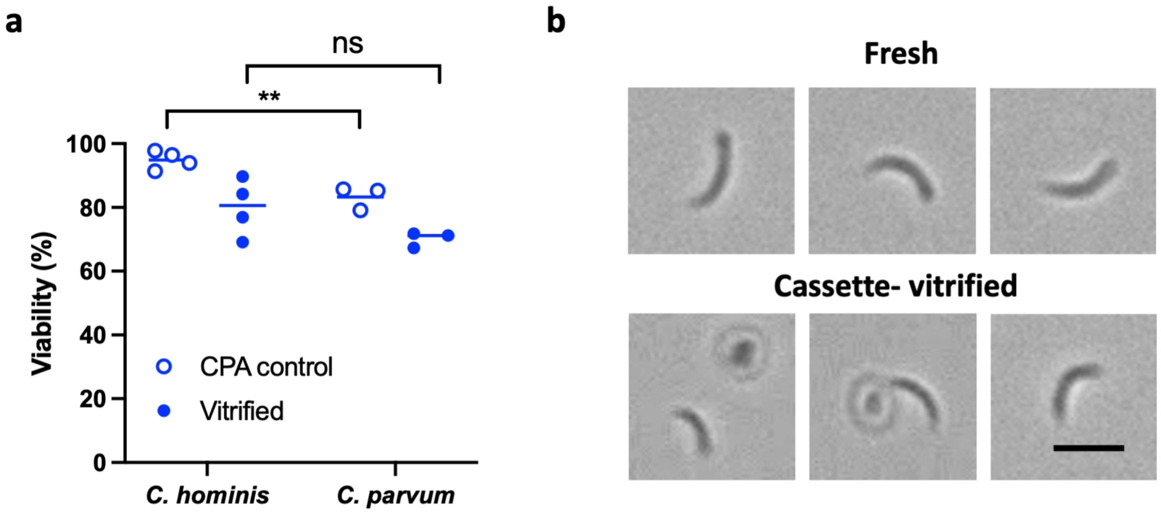

Supplementary Figure S11. Comparison of the cryoprotective protocol between *C. hominis* and *C. parvum* oocysts. One batch of 2-4-weeks-old oocysts was cryopreserved in cassettes using the 2 min protocol of 0.5 M trehalose/50% DMSO exposure at 37 °C. **a)** Oocyst viability was determined microscopically by means of PI exclusion, both before (CPA control) and after vitrification (vitrified). While a small difference in CPA toxicity was observed between species (p=0.005, t-test), there was no observed difference in after-thaw viability (p=0.12, t-test). Data points indicate individual values and lines represents the mean. **b)** Sporozoites of *C. parvum* excysted from oocysts vitrified in cassettes remain morphologically similar to fresh controls. Scale indicates 5 µm.
