# Supplementary material for: Scalable cryopreservation of infectious Cryptosporidium hominis oocysts by vitrification
Source: PLoS Pathog. 2023 Jun 8;19(6):e1011425. doi: 10.1371/journal.ppat.1011425 (PMC10284403; doi:10.1371/journal.ppat.1011425)
Supplement: S1 Protocol — (PDF) [file ppat.1011425.s013.pdf]

Supplementary Protocol S1. Rapid cooling of *C. hominis* oocysts using high aspect ratio cassettes. The method of ultra-fast cooling in high aspect ratio cassettes reported here was demonstrated to be a robust method to cryopreserve *C. hominis* oocysts using thermal permeabilization to DMSO at 37 °C. The method is however sensitive to variations in technique. A detailed protocol provided below discusses potential missteps. Special attention to safety is required when handling liquid nitrogen, including but not limited to the use of face shield and cryoprotective gloves.

Note: When planning for experiments, the oocysts input should be adjusted to account for a 45 % sample retention in the cassette and additional 25 % loss to lysis resulting from cryopreservation and thawing (Supplementary Figure S6). In consequence, the overall recovery of full oocysts from cassettes after cryopreservation is 30%, of which 70-75% are viable.

Materials:

- 1.5 mL microcentrifuge tubes- VWR, #10025-728
- Trehalose- Sigma, #T9531
- DMSO- Sigma, #D2650-5x5ml
- Cassette – Grace Bio-Labs, # RD500893
- 27 GA blunt needle- Sigma, #917532
- Self -clamping tweezers- Techni-Pro, #758TW402
- 50 mL conical centrifuge tubes
- 1x phosphate buffered saline
- Scissors

Protocol:

1. Approximately 2 million of >10-week-old *C. hominis* (Tu502 isolate) oocysts were centrifuged at 18,000xg for 2 min in 1.5 mL microcentrifuge tube. Supernatant was then completely removed in preparation for step #2. **Note:** To limit loss of oocysts during centrifugation, we recommend use of VWR polypropylene microcentrifuge tubes.

2. The packed oocyst pellet was suspended in 50 µL of 1 M trehalose solution (prepared in PBS) and incubated at ambient temperature for 10 min.

3. 50 µL of a 100% solution of DMSO pre-warmed at 37 °C was then added to the oocysts to achieve a final concentration of 0.5 M trehalose/50% DMSO and incubated in a 37 °C heat block for 2 min. Immediately after incubation is completed, oocysts are loaded into a cassette.

4. Cassette loading: 100 µL of oocysts in CPA cocktail was loaded through a cassette loading port using a 1 ml syringe affixed to a ≥ 27 G blunt needle by puncturing a silicone tab at an angle, while the tab at the opposite port is perforated with a needle to

allow for evacuation of displaced air, as demonstrated in Figure S3a. It is critical that loading is performed at ambient temperature and is completed within 1 min to minimize DMSO permeation and damage to oocysts. The cassette was placed on a clean Petri dish during loading.

5. Freezing: Using tweezers, the cassette was placed above Styrofoam box filled with liquid nitrogen perpendicular to the surface of liquid. Using a steady but rapid movement, the cassette was plunged into the liquid nitrogen. Rapid submersion ensures even cooling rates across the sample and is likely critical to achieve the positive cryopreservation outcome.

6. Thawing: Using tweezers, the cassette was transferred into a 40 ºC water bath in a steady but rapid movement and allowed to thaw for 10 sec.

   Note: Based on our experience with other applications, variations in technique during cassette loading, freezing and thawing steps are the most common reasons for poor cryopreservation outcome.

7. Sample recovery: The cassette was dried with a tissue wipe and placed on a clean Petri dish. Two corners of cassette were cut off using scissors to expose exit channels. The cassette was then placed in 50 mL centrifuge tube filled with 3 mL of PBS and centrifuged at 200 ×g for 1 min to expel contents directly into PBS.

8. The cassette was discarded, and additional 2 mL of PBS was added to the solution containing expelled oocysts. Oocyst solution was then incubated at ambient temperature for 30 min to allow DMSO to diffuse out from oocyst.

9. 5 mL of oocyst solution was then centrifuged, either in 50 mL tube at 2,500 ×g, 10 min or repeatedly in 1.5 mL microcentrifuge tube at 18,000 ×g, 2 min.

10. The supernatant was removed, and oocysts were resuspended in 100 µL PBS, followed by evaluation of viability and excystation. For evaluation of infectivity in gnotobiotic piglets, oocysts were suspended in PBS containing penicillin (100 U/mL) and streptomycin (100 µg/mL).
