# Supplementary figures and images for: Scalable cryopreservation of infectious Cryptosporidium hominis oocysts by vitrification
Source: PLoS Pathog. 2023 Jun 8;19(6):e1011425. doi: 10.1371/journal.ppat.1011425 (PMC10284403; doi:10.1371/journal.ppat.1011425)

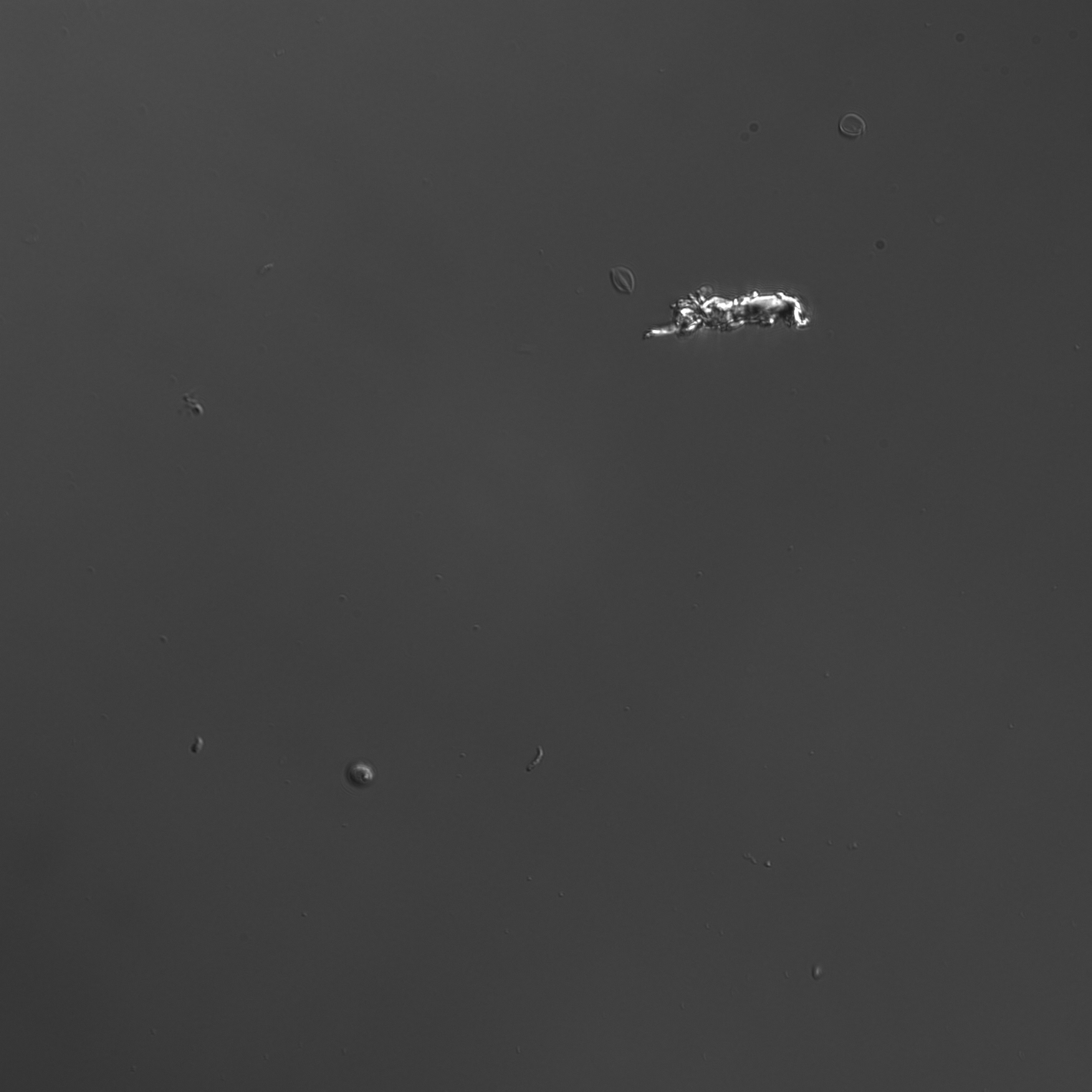

Supplement: S1 Data — (ZIP) [file ppat.1011425.s014.zip › Supporting Information Data/micrographs/Fig. 3c/PBS-frozen (top right).jpg]

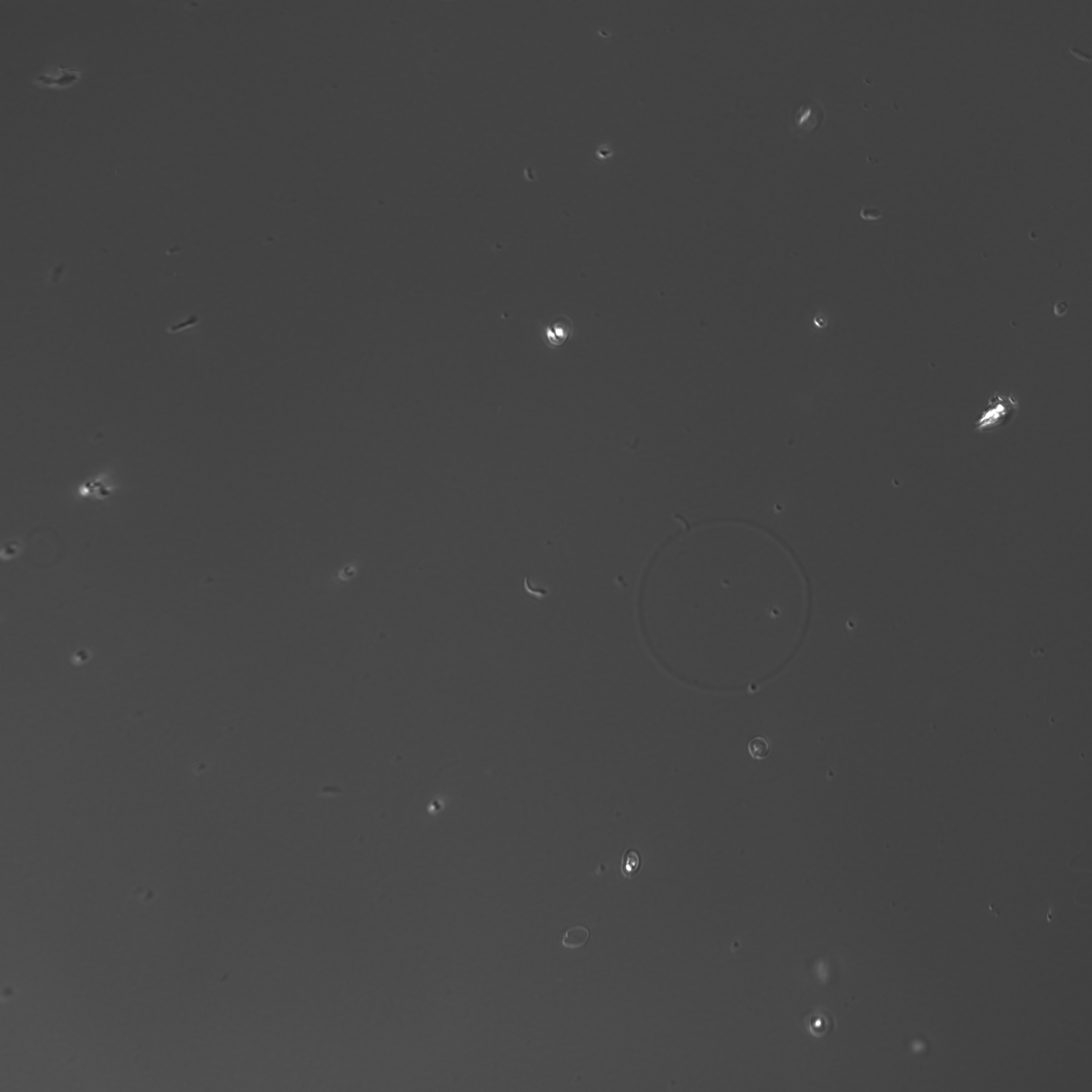

Supplement: S1 Data — (ZIP) [file ppat.1011425.s014.zip › Supporting Information Data/micrographs/Fig. 3c/┬╡capillary-vitrified (top middle) .tif]

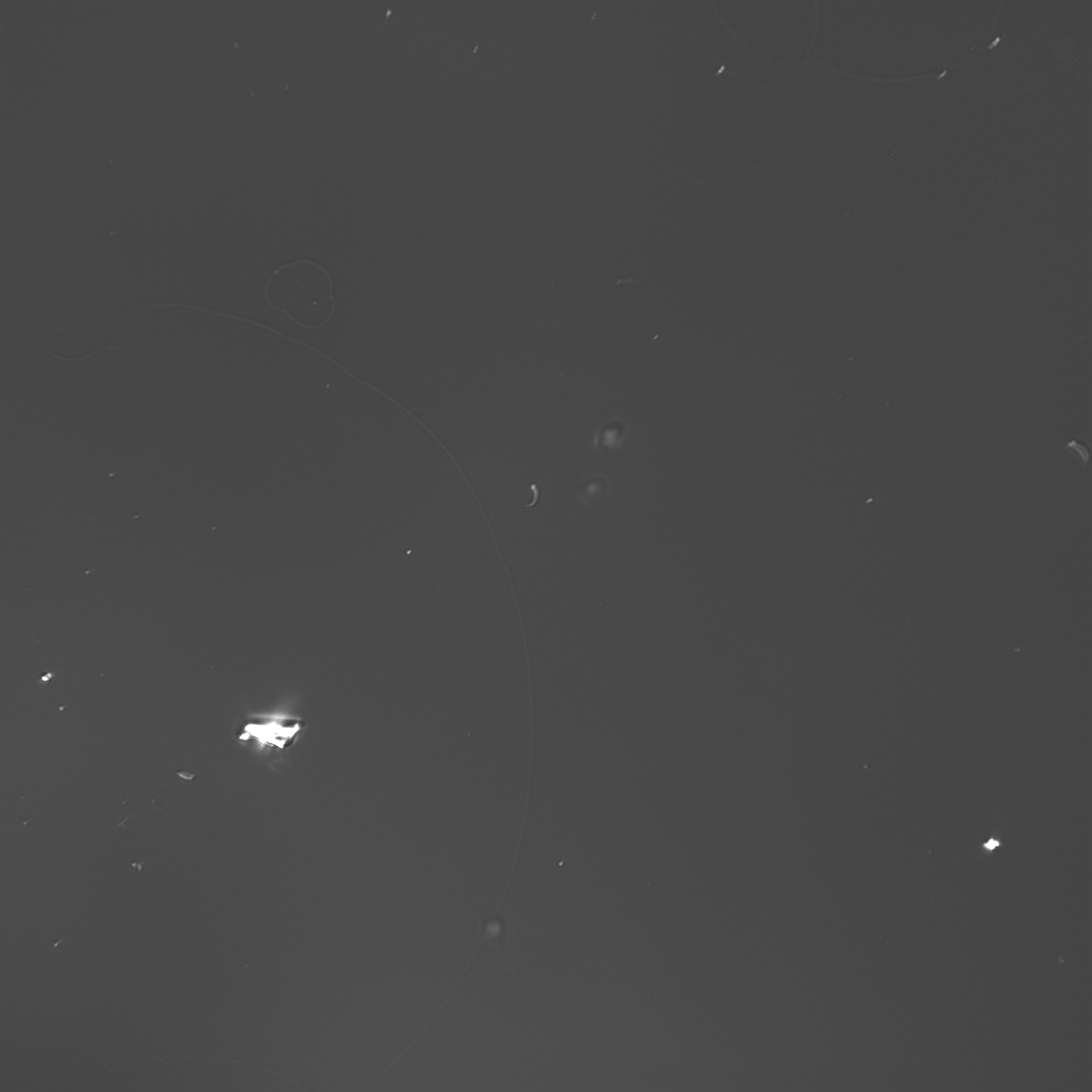

Supplement: S1 Data — (ZIP) [file ppat.1011425.s014.zip › Supporting Information Data/micrographs/Fig. 3c/┬╡capillary-vitrified (bottom middle) .tif]

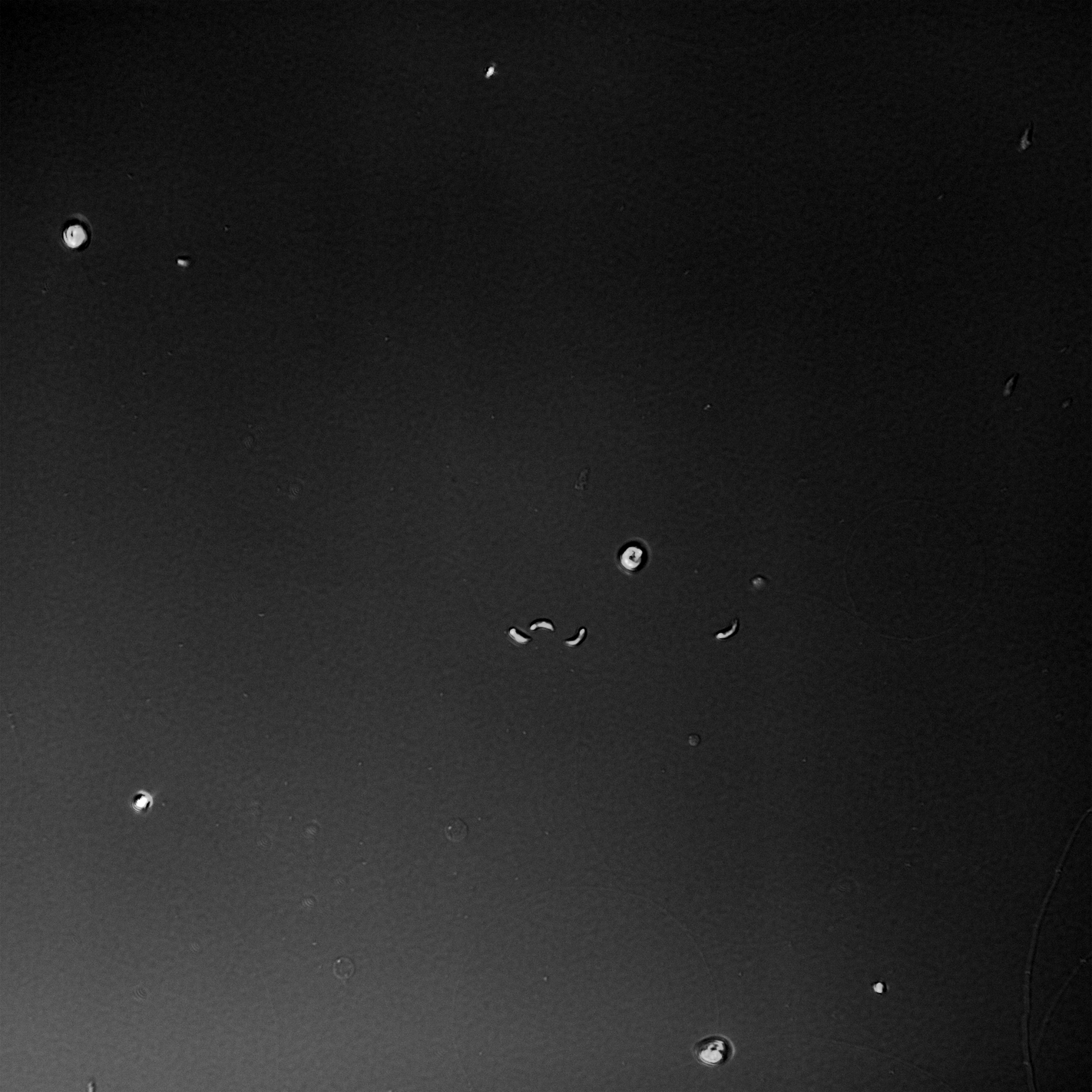

Supplement: S1 Data — (ZIP) [file ppat.1011425.s014.zip › Supporting Information Data/micrographs/Fig. 3c/Fresh control (bottom left).tif]

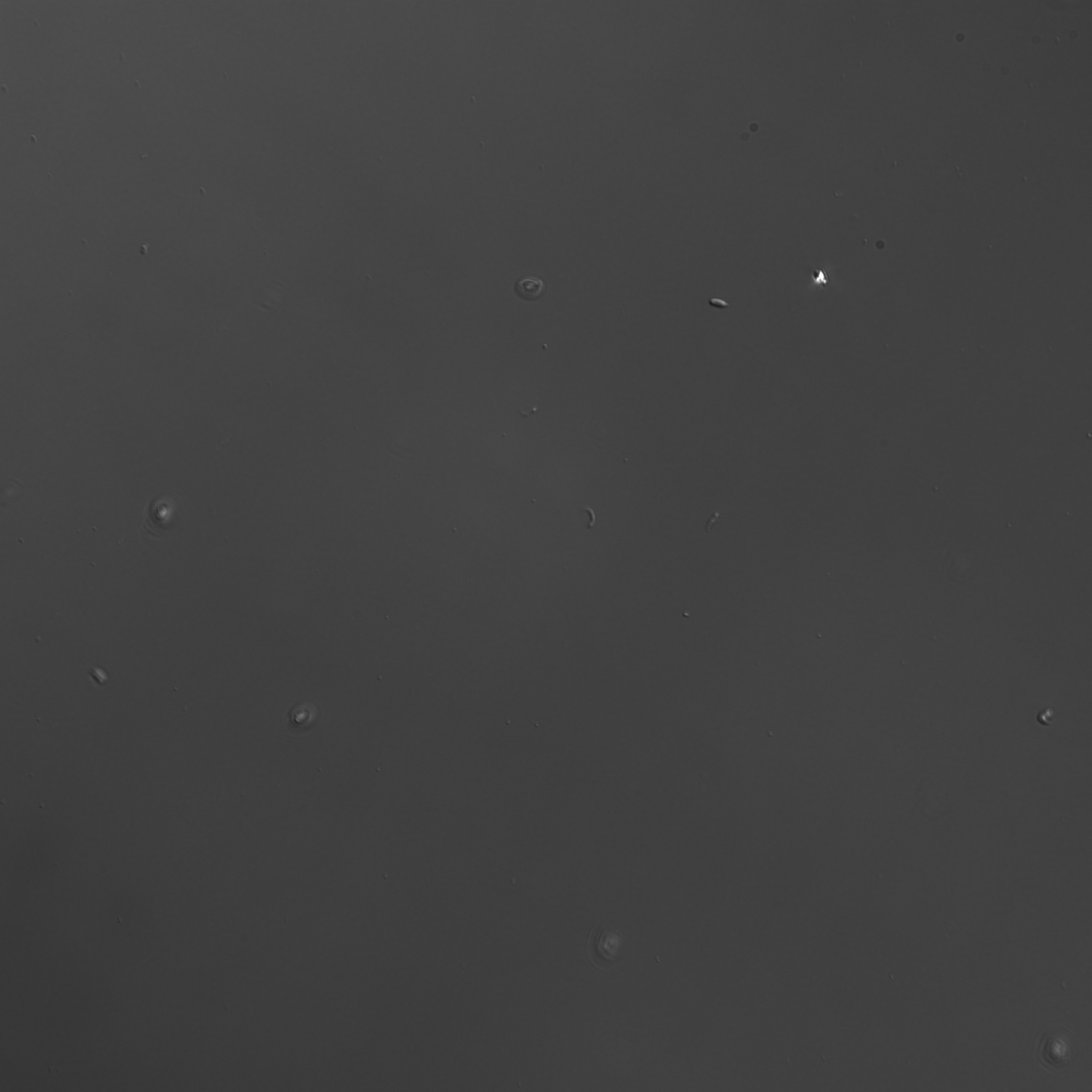

Supplement: S1 Data — (ZIP) [file ppat.1011425.s014.zip › Supporting Information Data/micrographs/Fig. 3c/PBS-frozen (bottom right).jpg]

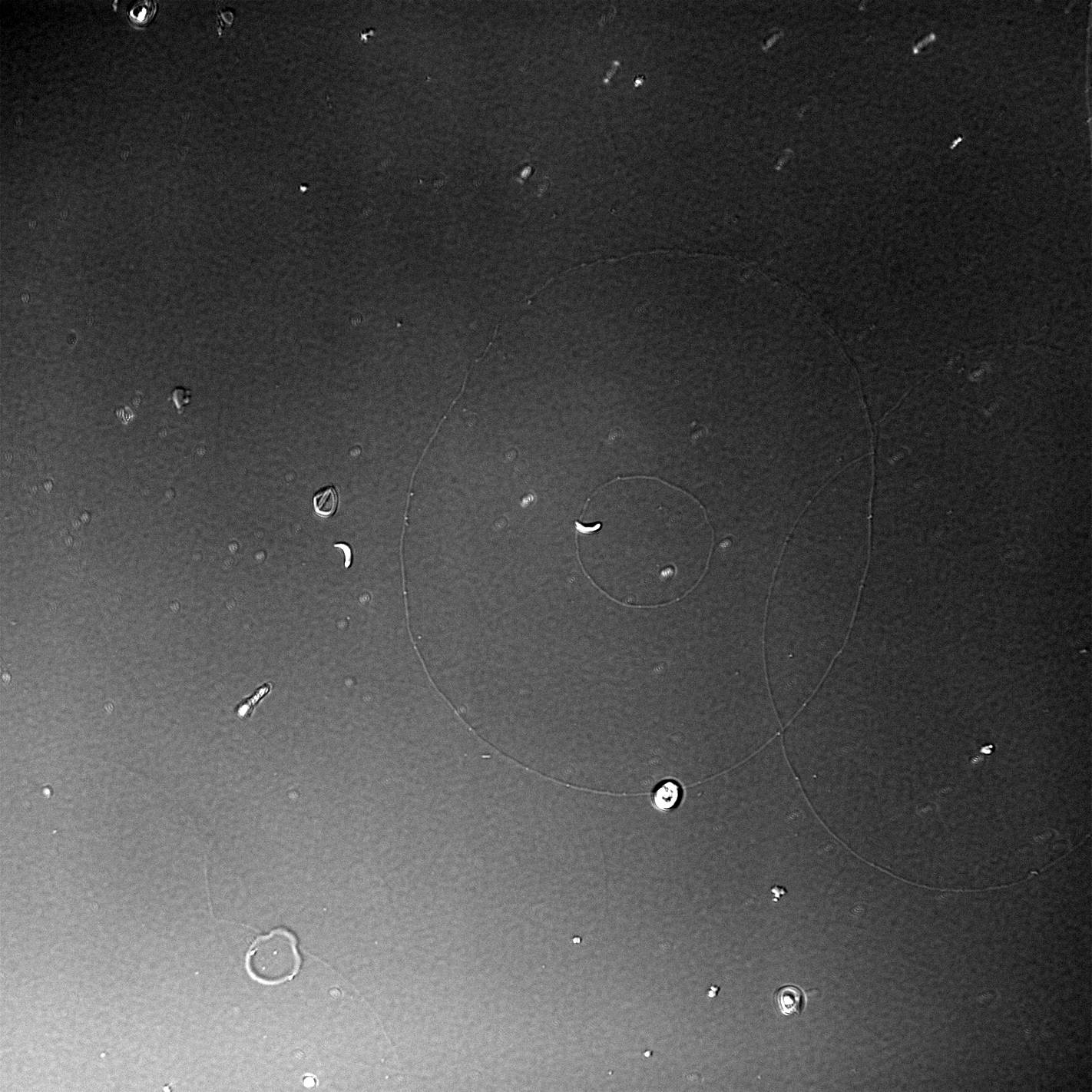

Supplement: S1 Data — (ZIP) [file ppat.1011425.s014.zip › Supporting Information Data/micrographs/Fig. 3c/Fresh control (top left).tif]

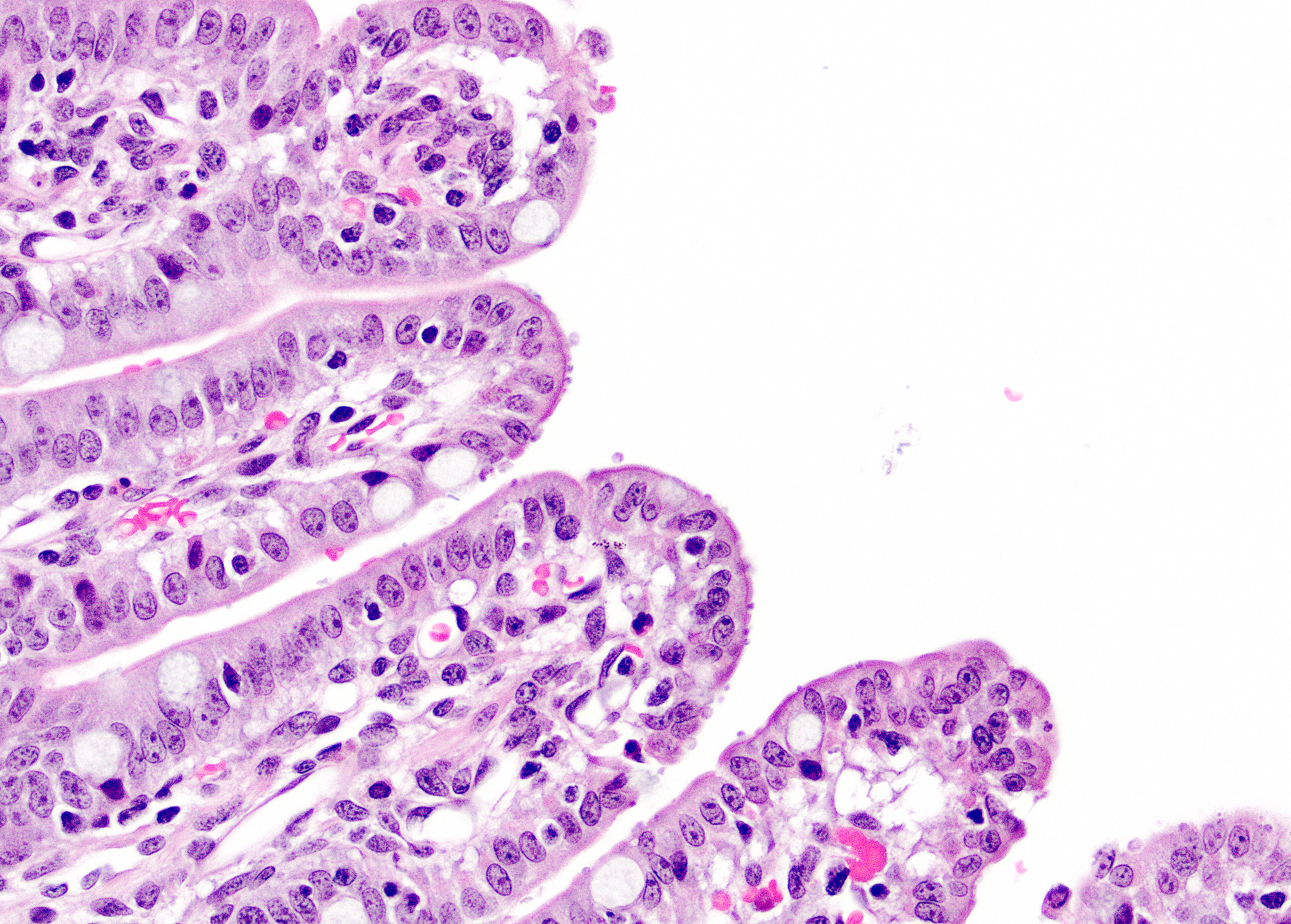

Supplement: S1 Data — (ZIP) [file ppat.1011425.s014.zip › Supporting Information Data/micrographs/Fig. 3e/40 month storage, pig 8, image 5.tiff]

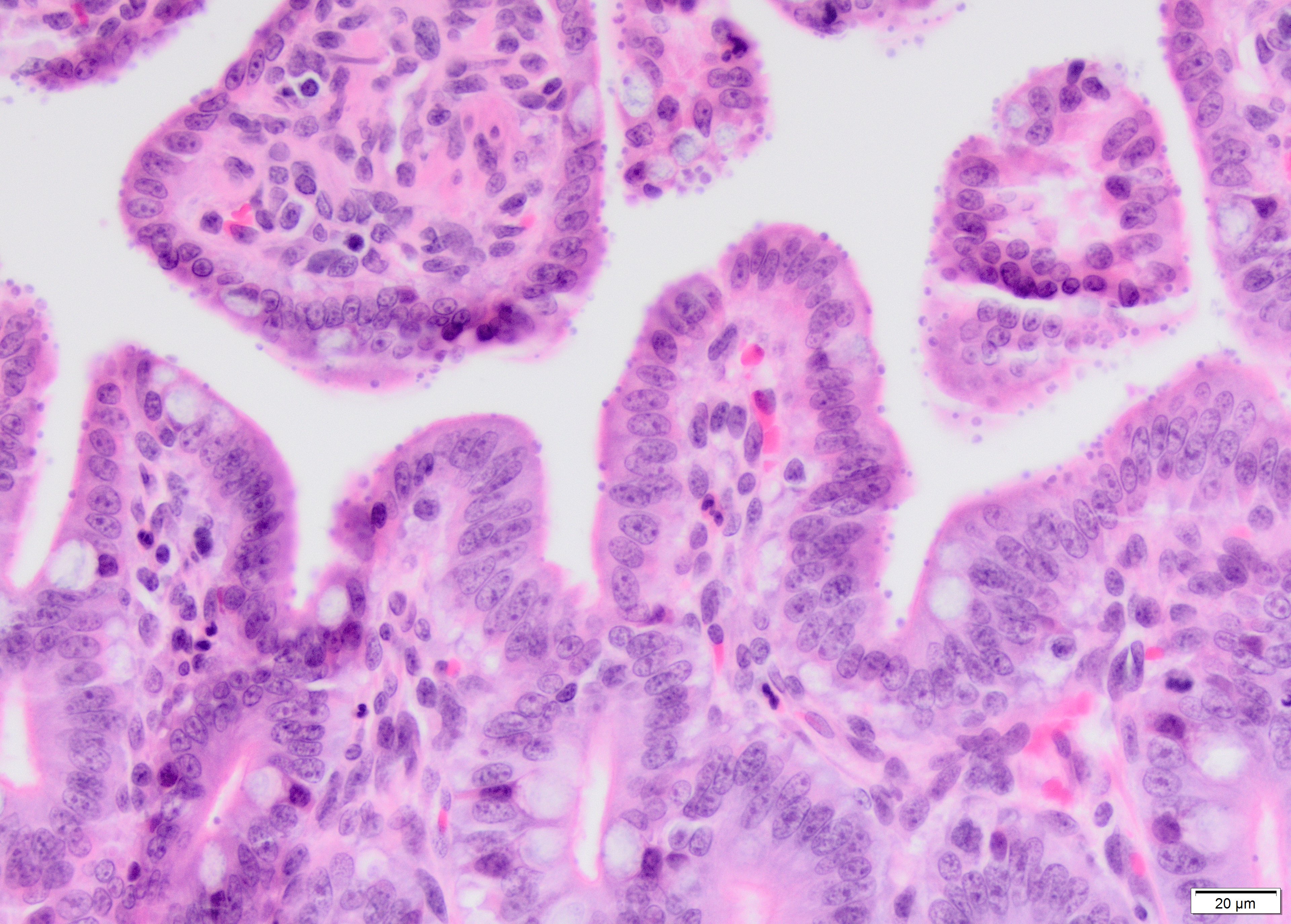

Supplement: S1 Data — (ZIP) [file ppat.1011425.s014.zip › Supporting Information Data/micrographs/Fig. 3e/fresh control (top).jpg]

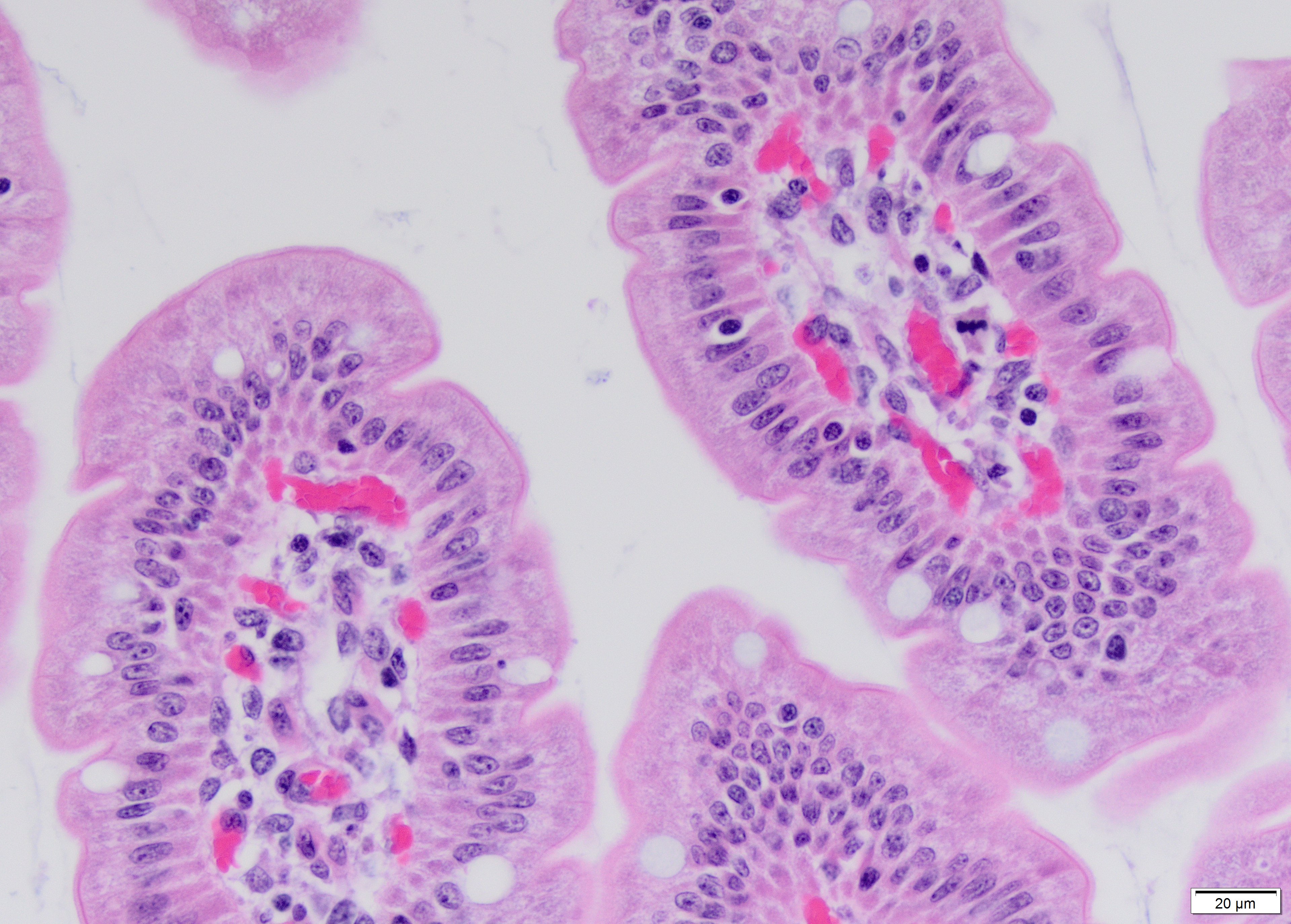

Supplement: S1 Data — (ZIP) [file ppat.1011425.s014.zip › Supporting Information Data/micrographs/Fig. 3e/┬╡capillary-vitrified (middle).jpg]

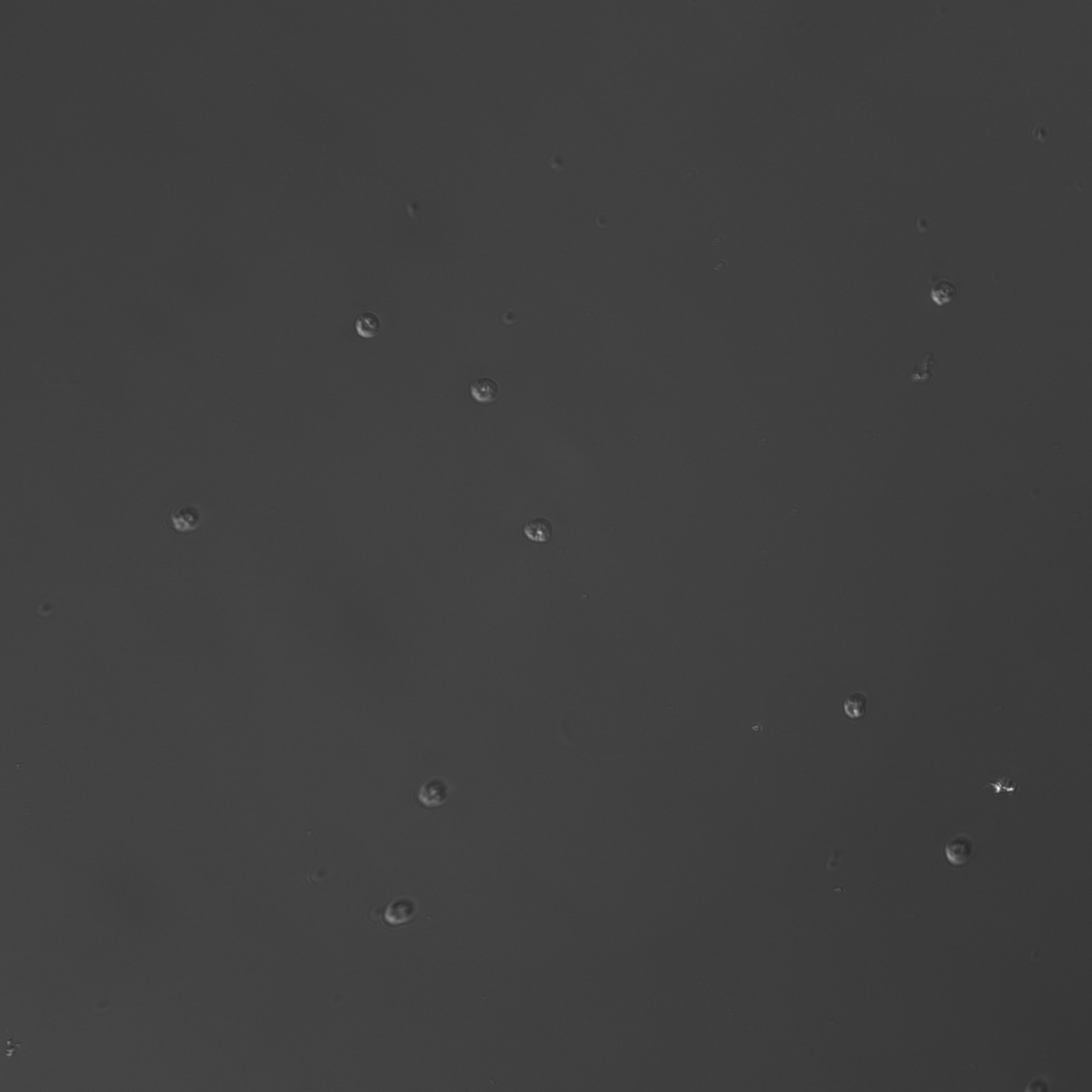

Supplement: S1 Data — (ZIP) [file ppat.1011425.s014.zip › Supporting Information Data/micrographs/Fig. 1c/PBS (bottom left).jpg]

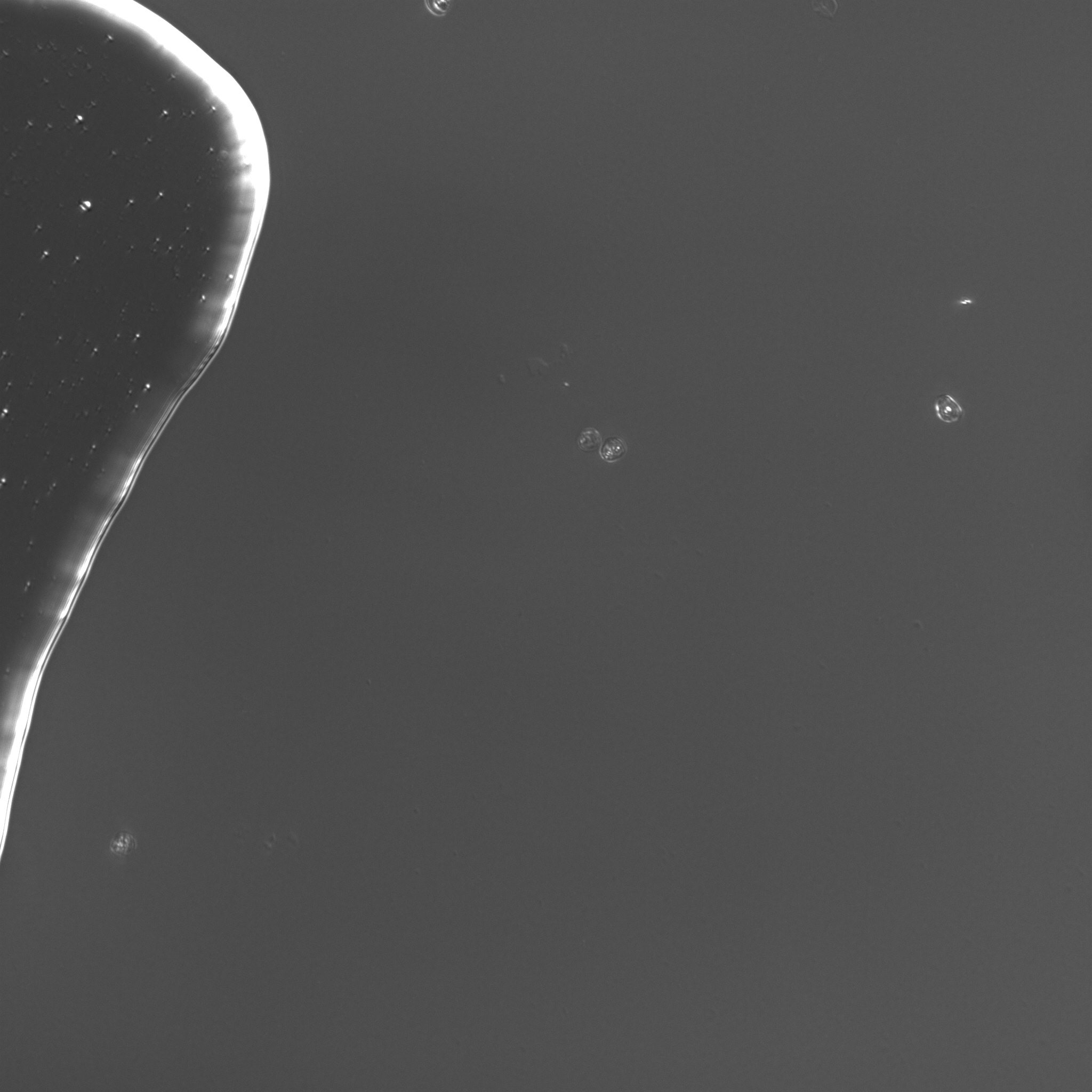

Supplement: S1 Data — (ZIP) [file ppat.1011425.s014.zip › Supporting Information Data/micrographs/Fig. 1c/trehalose (top middle).jpg]

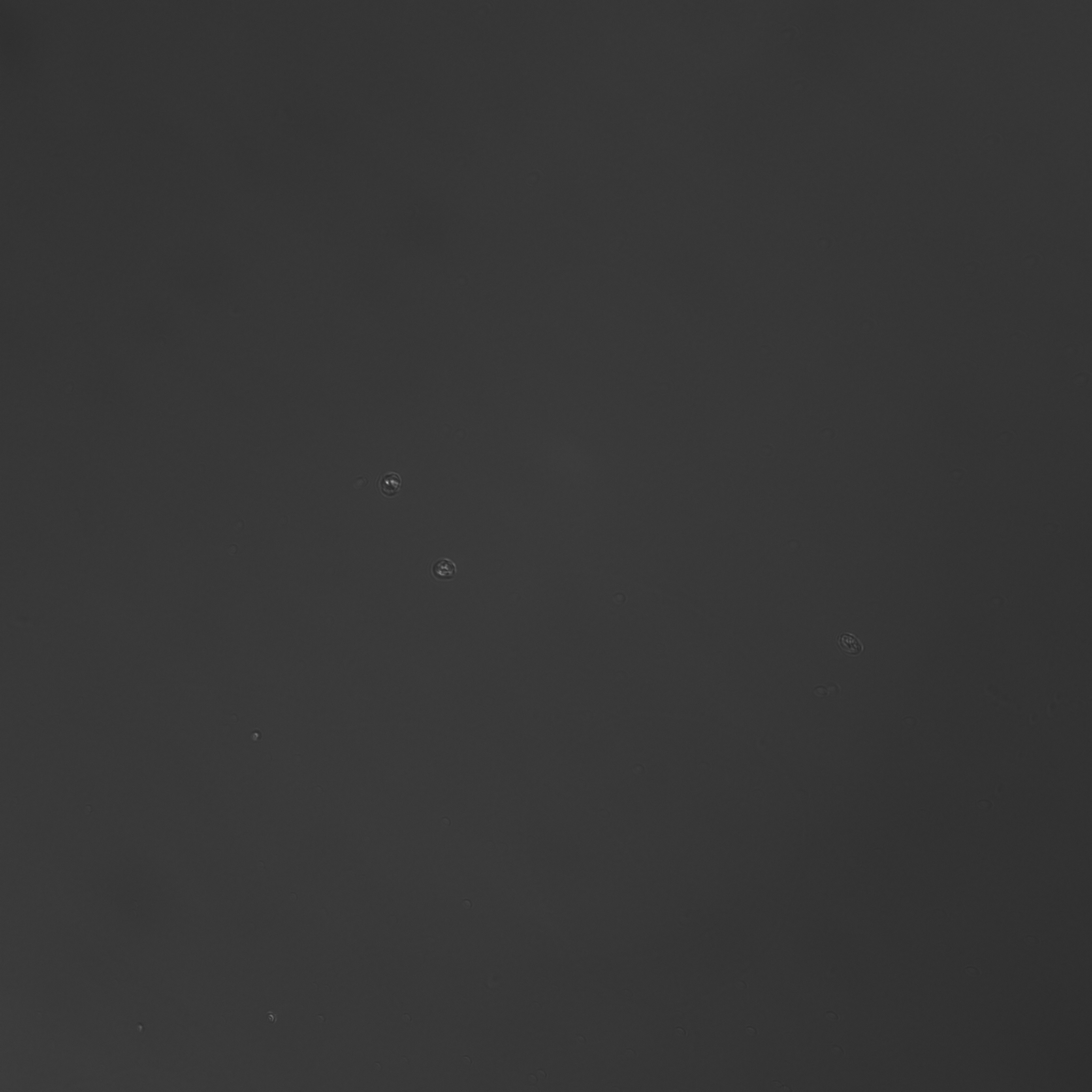

Supplement: S1 Data — (ZIP) [file ppat.1011425.s014.zip › Supporting Information Data/micrographs/Fig. 1c/trehalose (bottom middle).jpg]

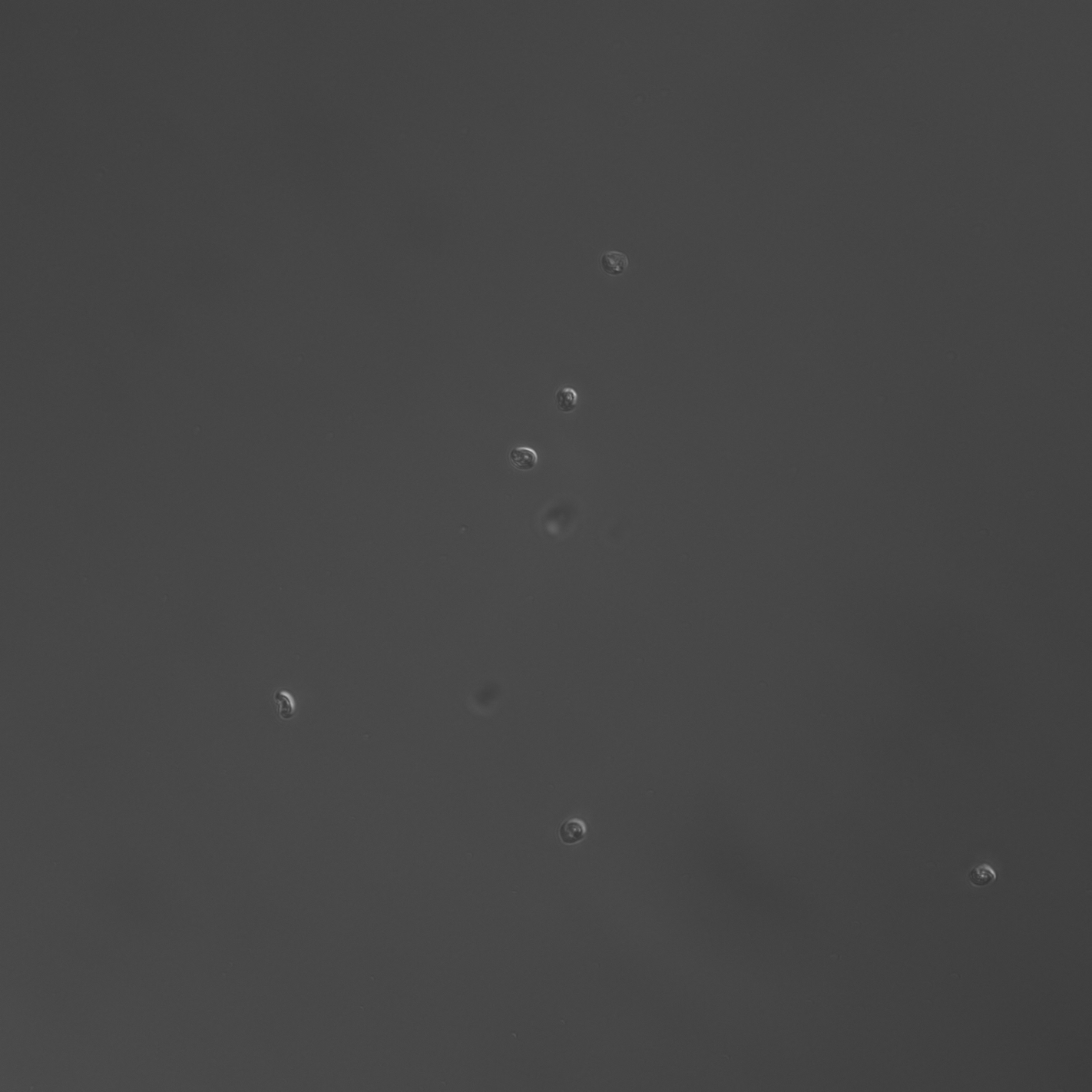

Supplement: S1 Data — (ZIP) [file ppat.1011425.s014.zip › Supporting Information Data/micrographs/Fig. 1c/DMSO (top right).jpg]

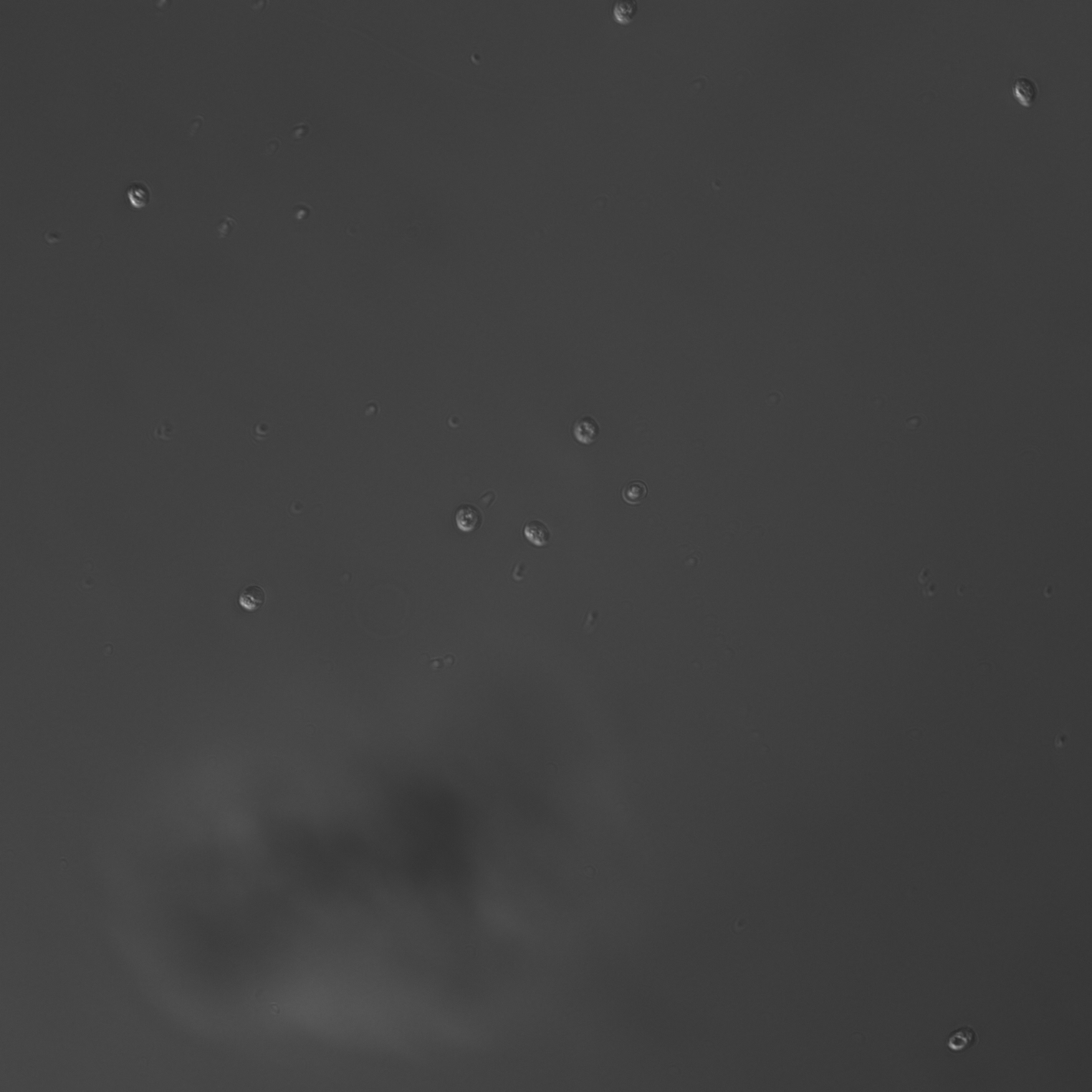

Supplement: S1 Data — (ZIP) [file ppat.1011425.s014.zip › Supporting Information Data/micrographs/Fig. 1c/PBS (top left).jpg]

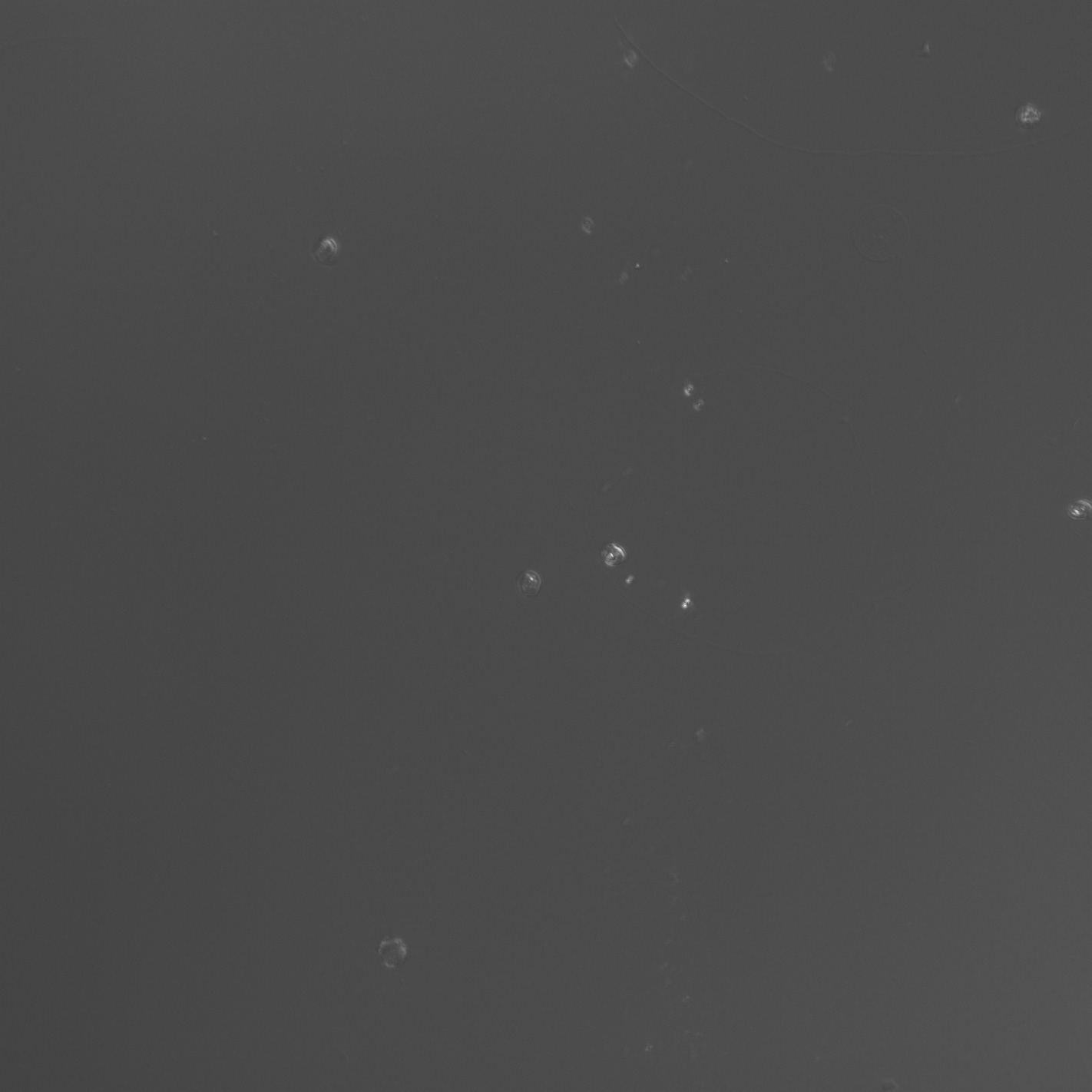

Supplement: S1 Data — (ZIP) [file ppat.1011425.s014.zip › Supporting Information Data/micrographs/Fig. 1c/PBS (bottom right).jpg]

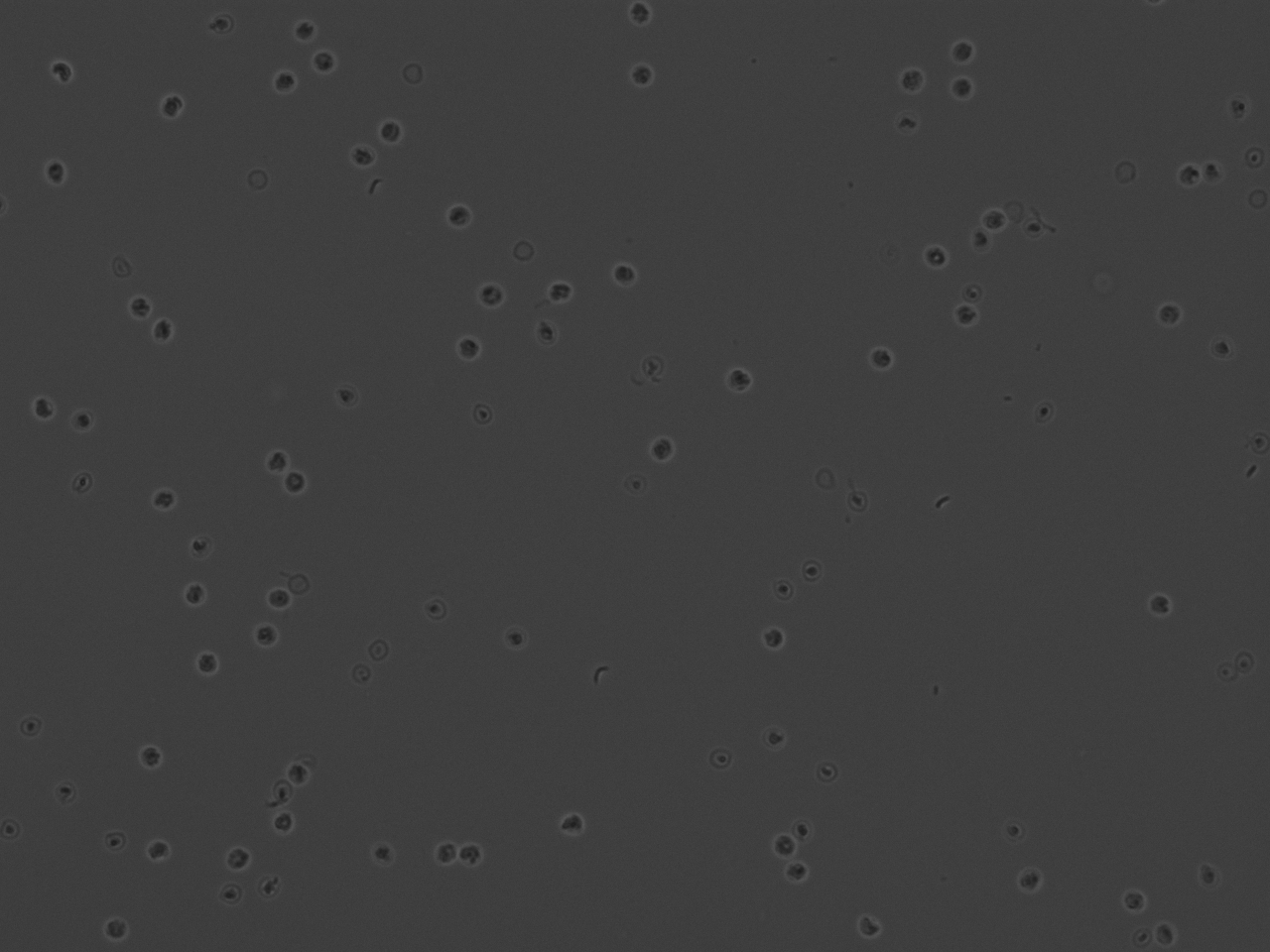

Supplement: S1 Data — (ZIP) [file ppat.1011425.s014.zip › Supporting Information Data/micrographs/Fig. S11/Cassette-vitrified (bottom right).tif]

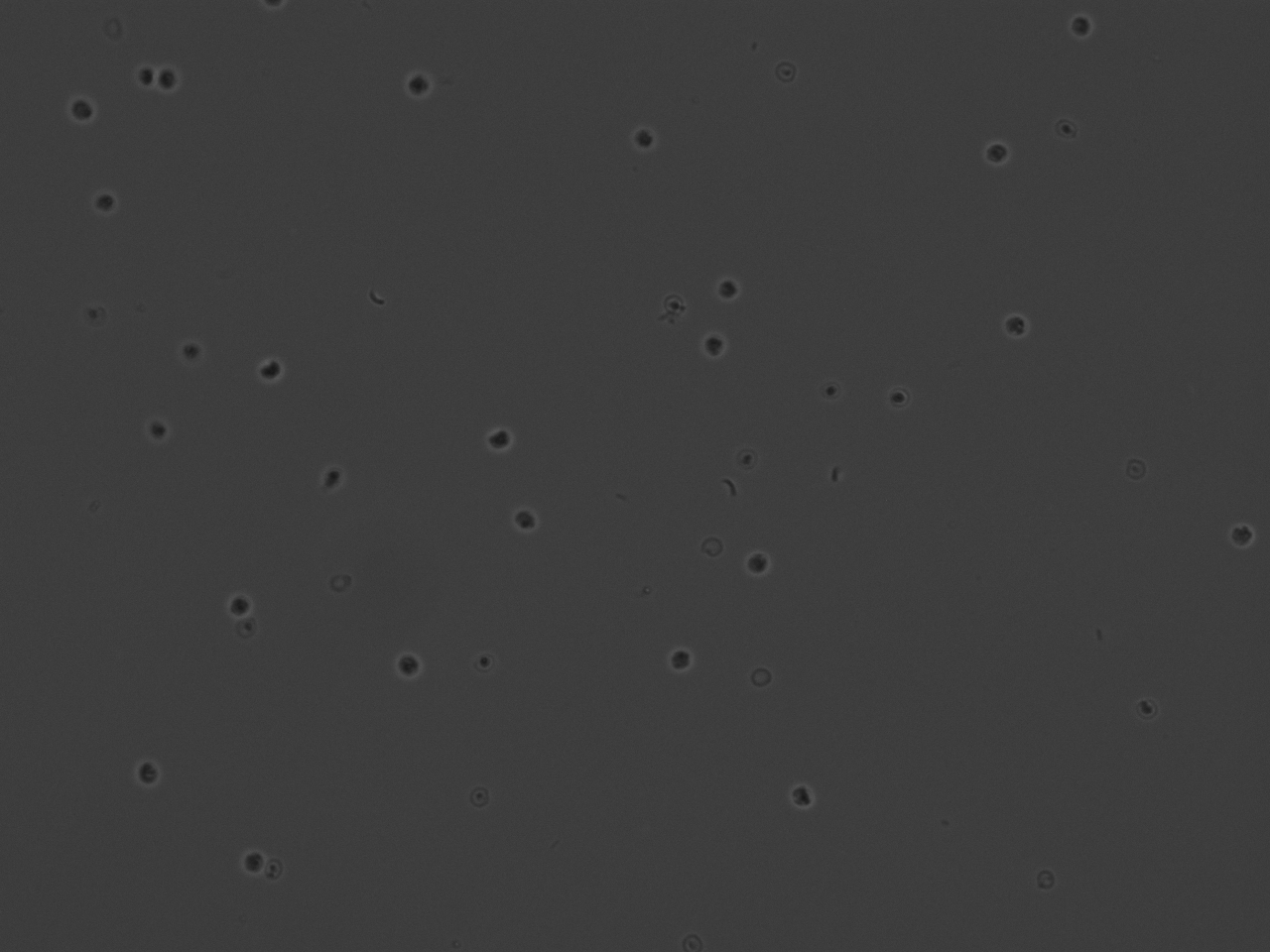

Supplement: S1 Data — (ZIP) [file ppat.1011425.s014.zip › Supporting Information Data/micrographs/Fig. S11/Cassette-vitrified (bottom left).tif]

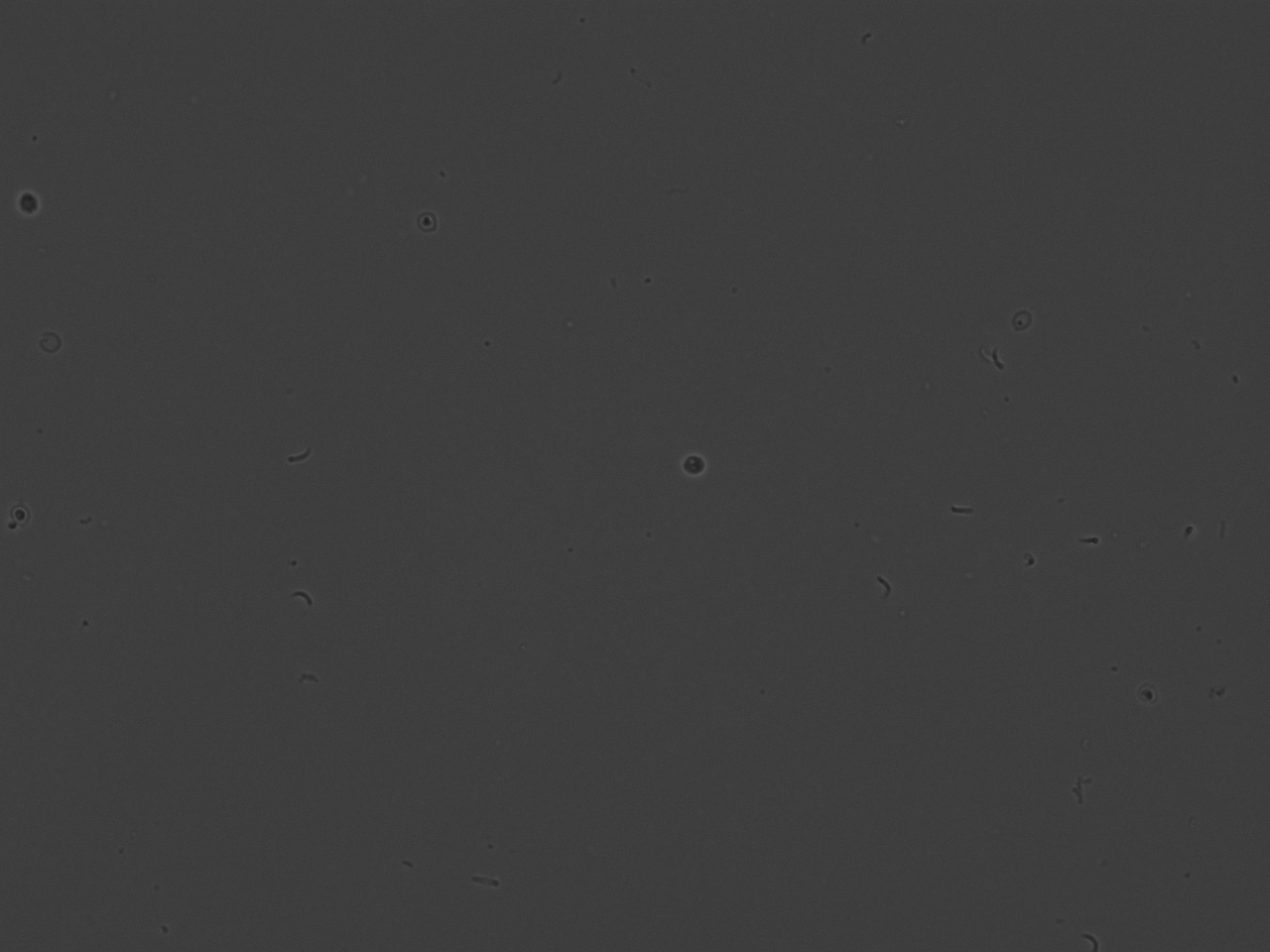

Supplement: S1 Data — (ZIP) [file ppat.1011425.s014.zip › Supporting Information Data/micrographs/Fig. S11/Fresh (top middle).tif]

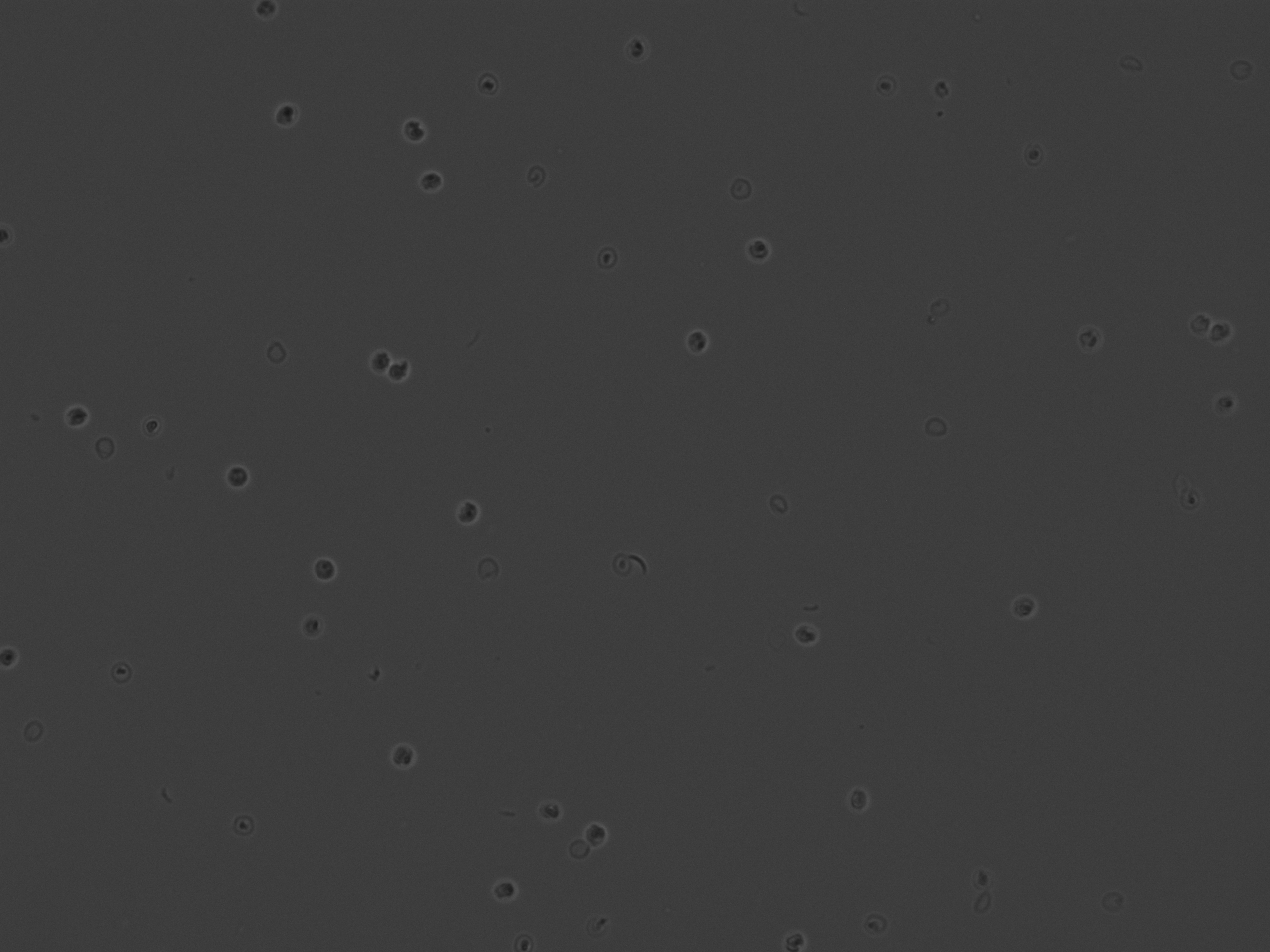

Supplement: S1 Data — (ZIP) [file ppat.1011425.s014.zip › Supporting Information Data/micrographs/Fig. S11/Cassette-vitrified (bottom middle).tif]

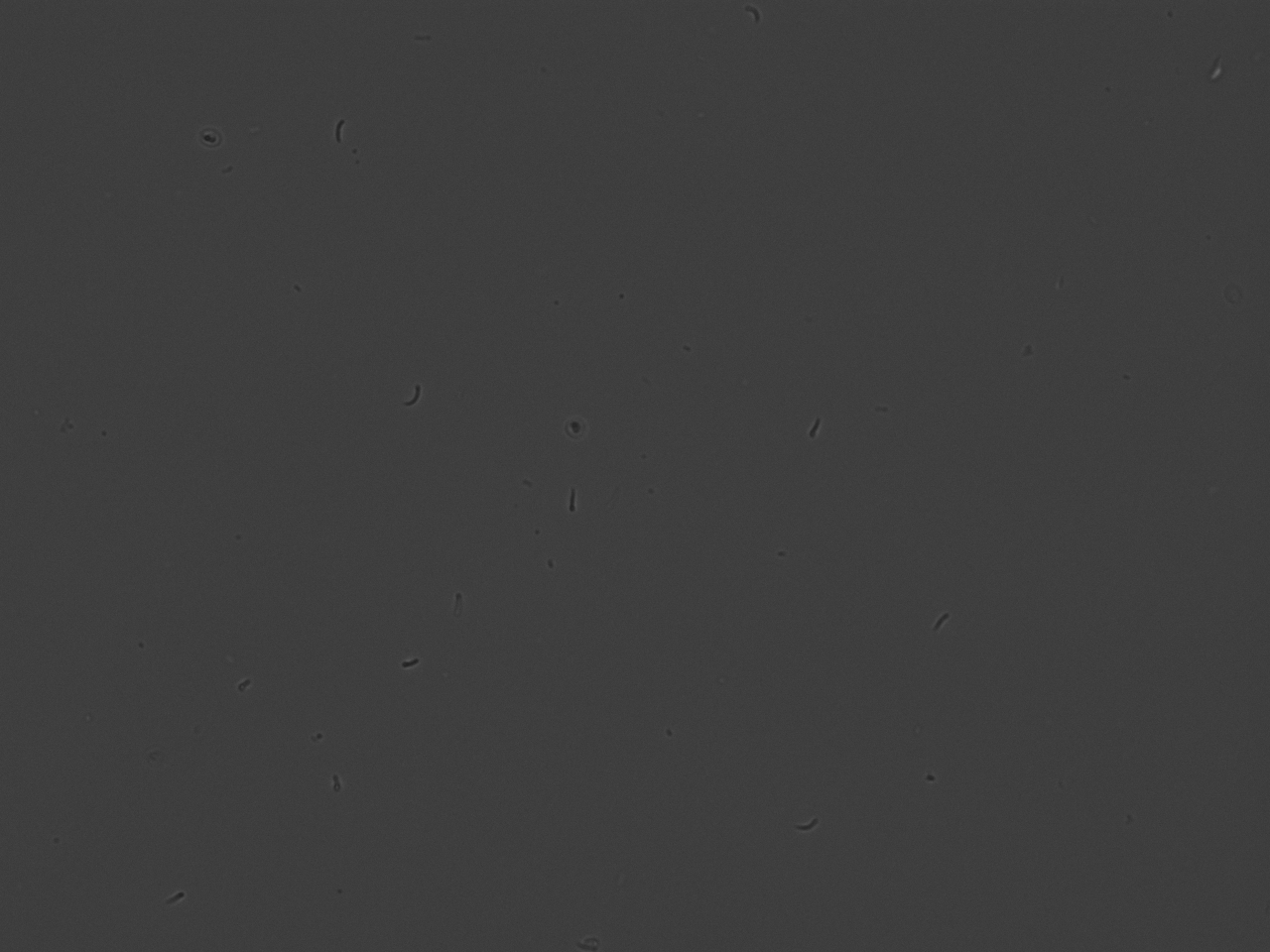

Supplement: S1 Data — (ZIP) [file ppat.1011425.s014.zip › Supporting Information Data/micrographs/Fig. S11/Fresh (top right).tif]

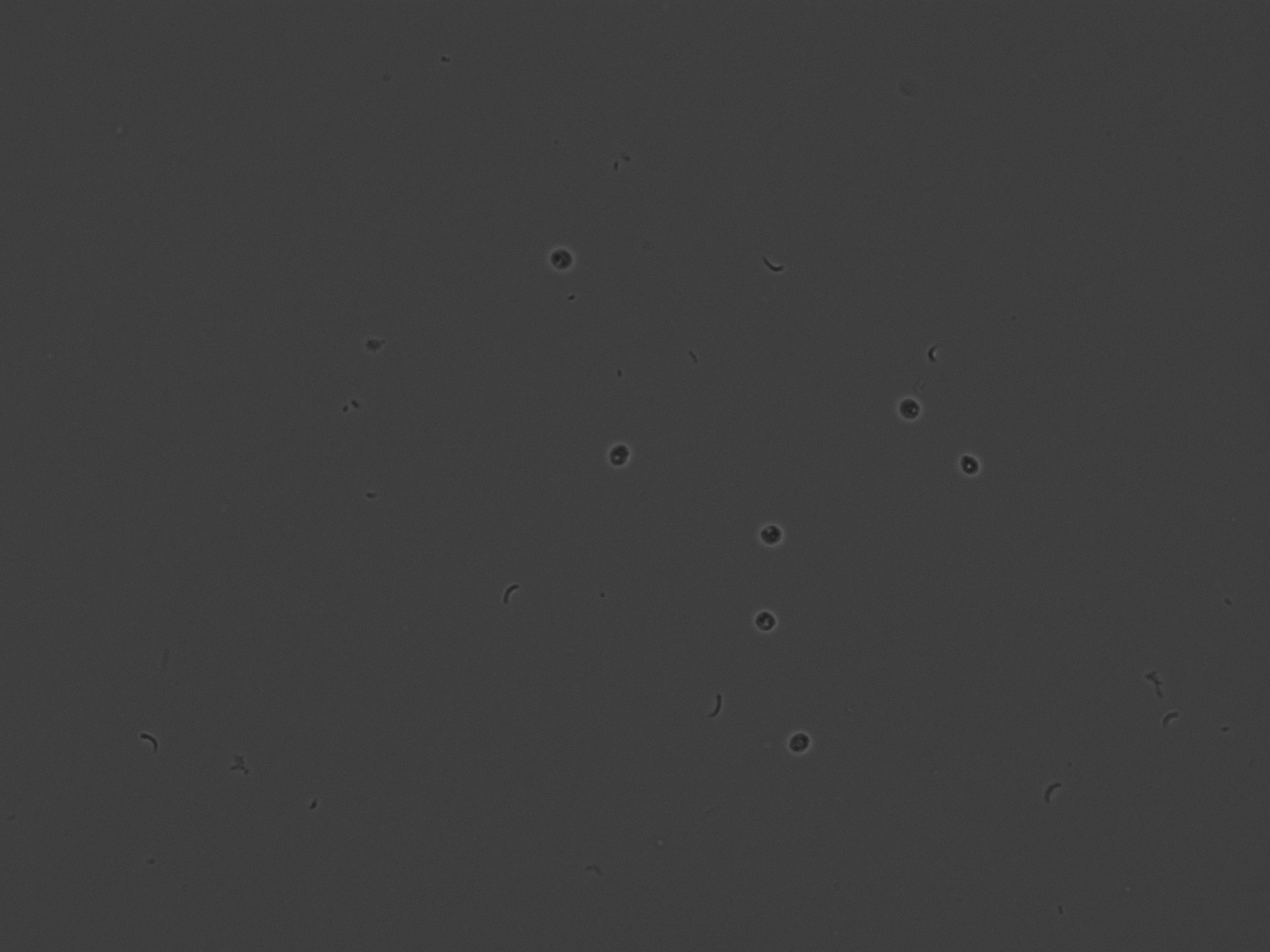

Supplement: S1 Data — (ZIP) [file ppat.1011425.s014.zip › Supporting Information Data/micrographs/Fig. S11/Fresh (top left).tif]

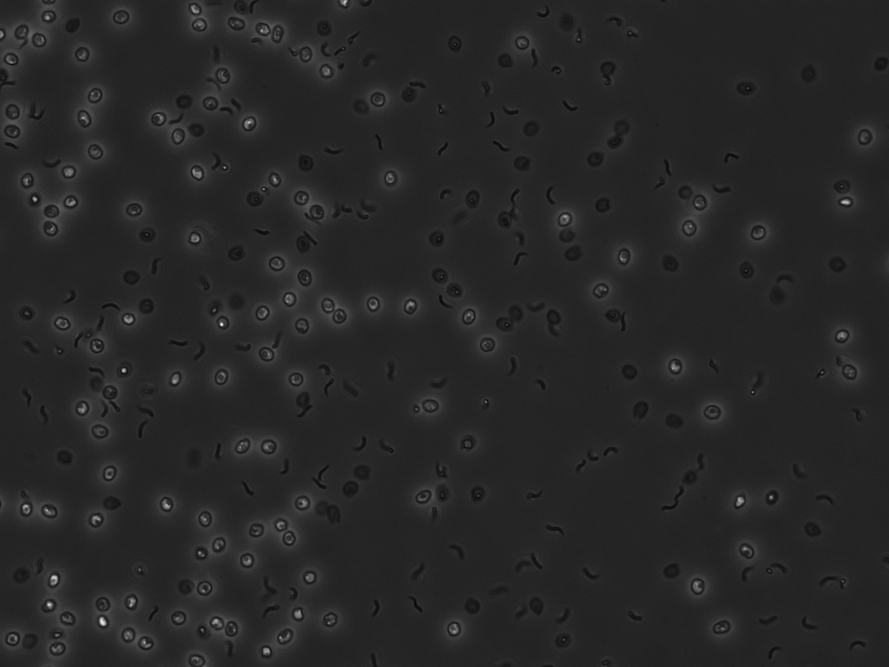

Supplement: S1 Data — (ZIP) [file ppat.1011425.s014.zip › Supporting Information Data/micrographs/Fig. 2d/Untreated oocysts (left).tif]

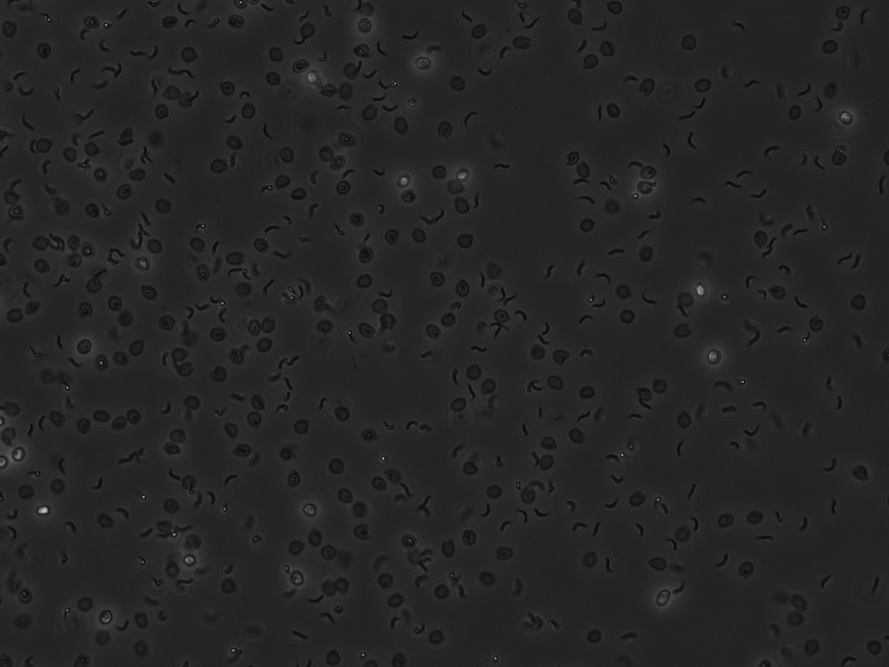

Supplement: S1 Data — (ZIP) [file ppat.1011425.s014.zip › Supporting Information Data/micrographs/Fig. 2d/DMSO-treated oocysts (right).tif]

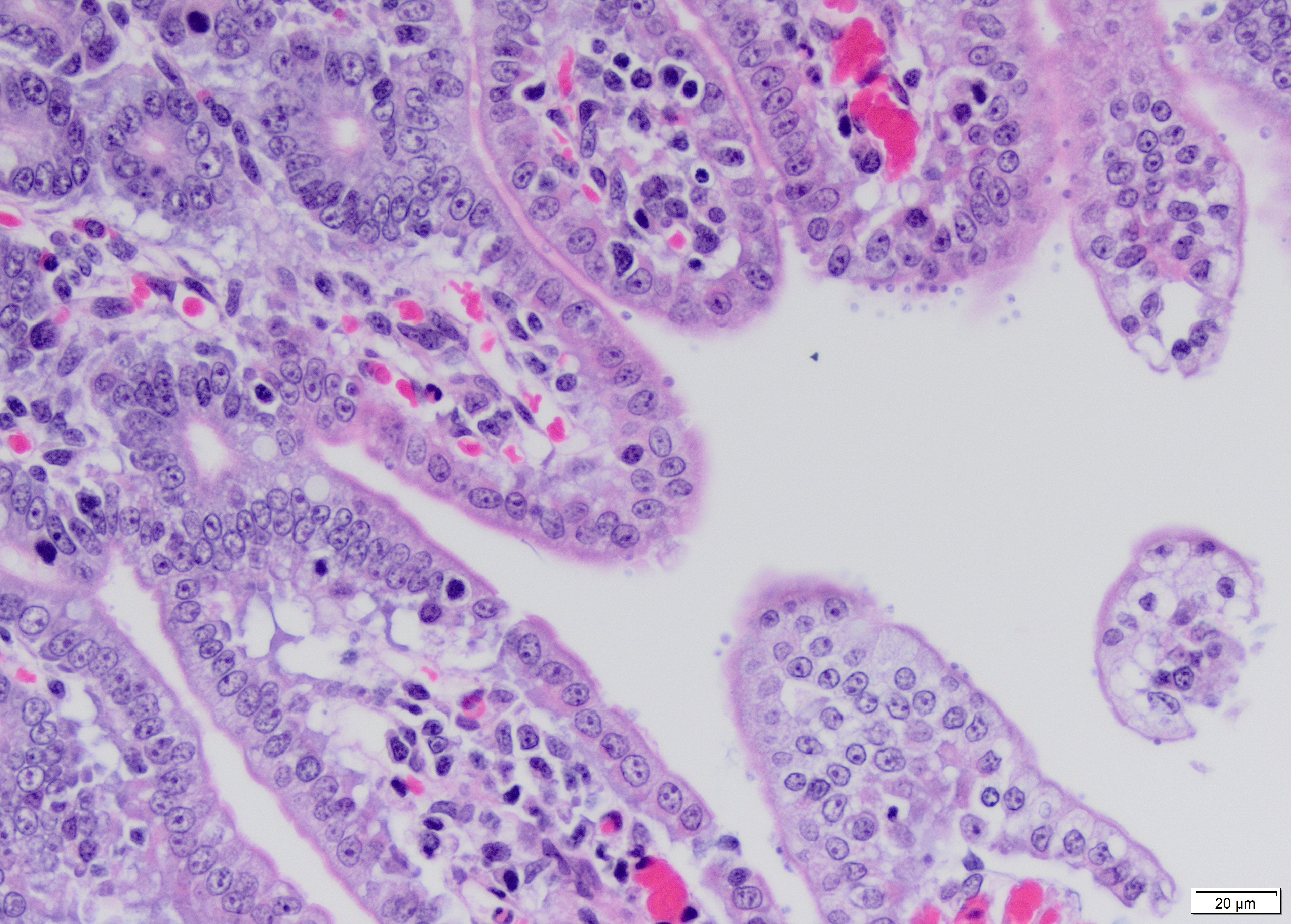

Supplement: S1 Data — (ZIP) [file ppat.1011425.s014.zip › Supporting Information Data/micrographs/Fig. S9/Cassette-vitrified (bottom left).jpg]

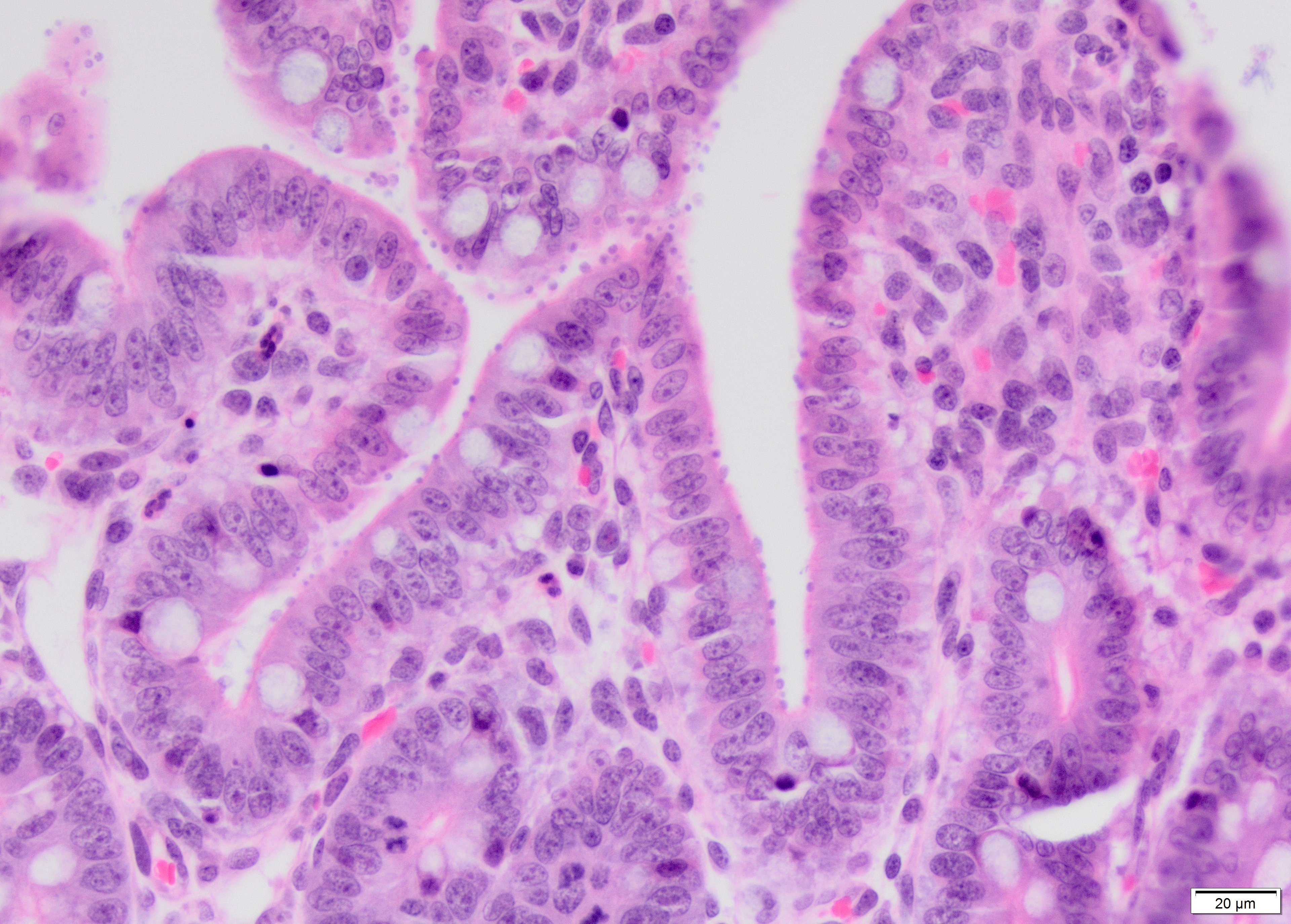

Supplement: S1 Data — (ZIP) [file ppat.1011425.s014.zip › Supporting Information Data/micrographs/Fig. S9/Positive control (top left).jpg]

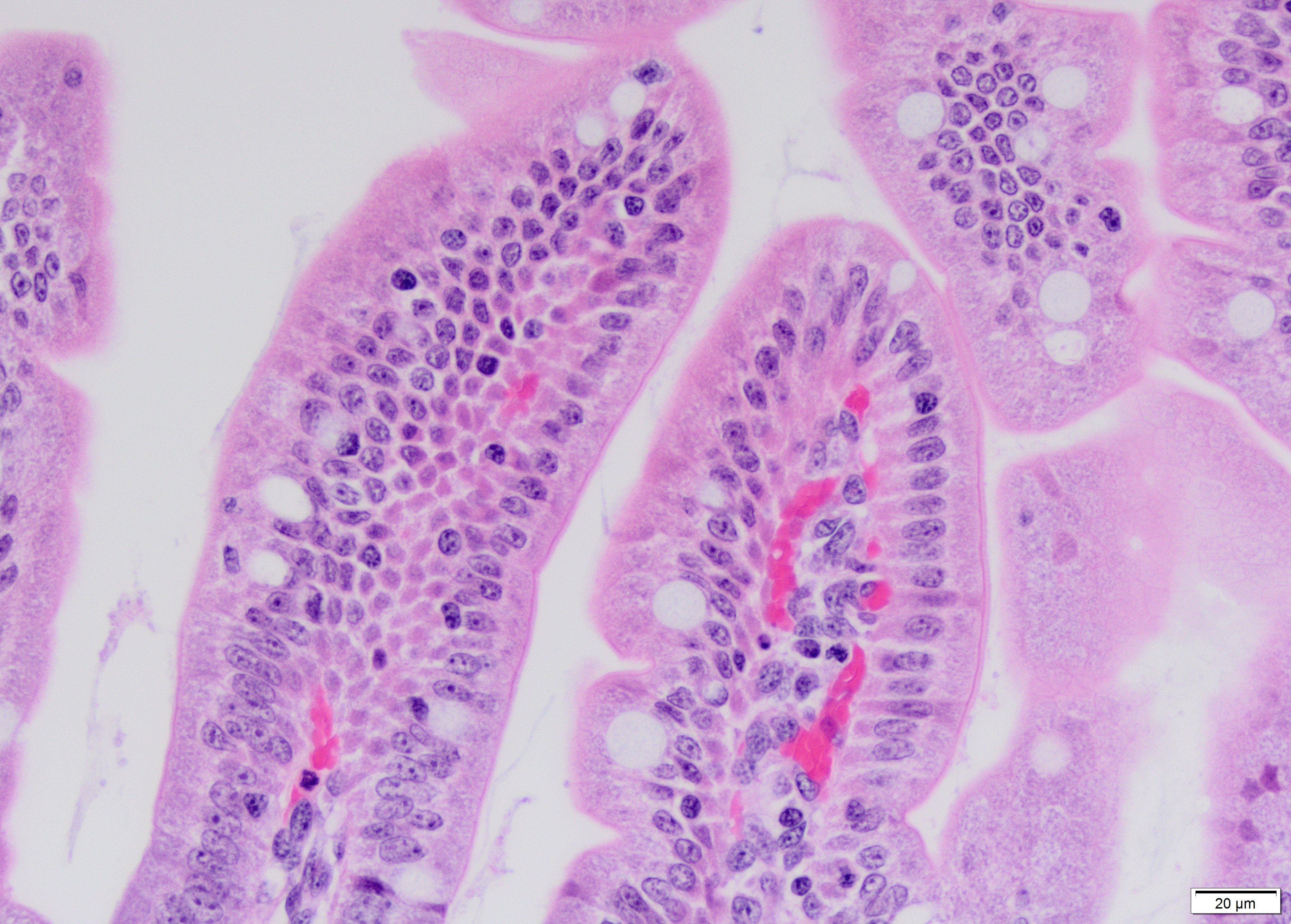

Supplement: S1 Data — (ZIP) [file ppat.1011425.s014.zip › Supporting Information Data/micrographs/Fig. S9/Negative control (top right).jpg]

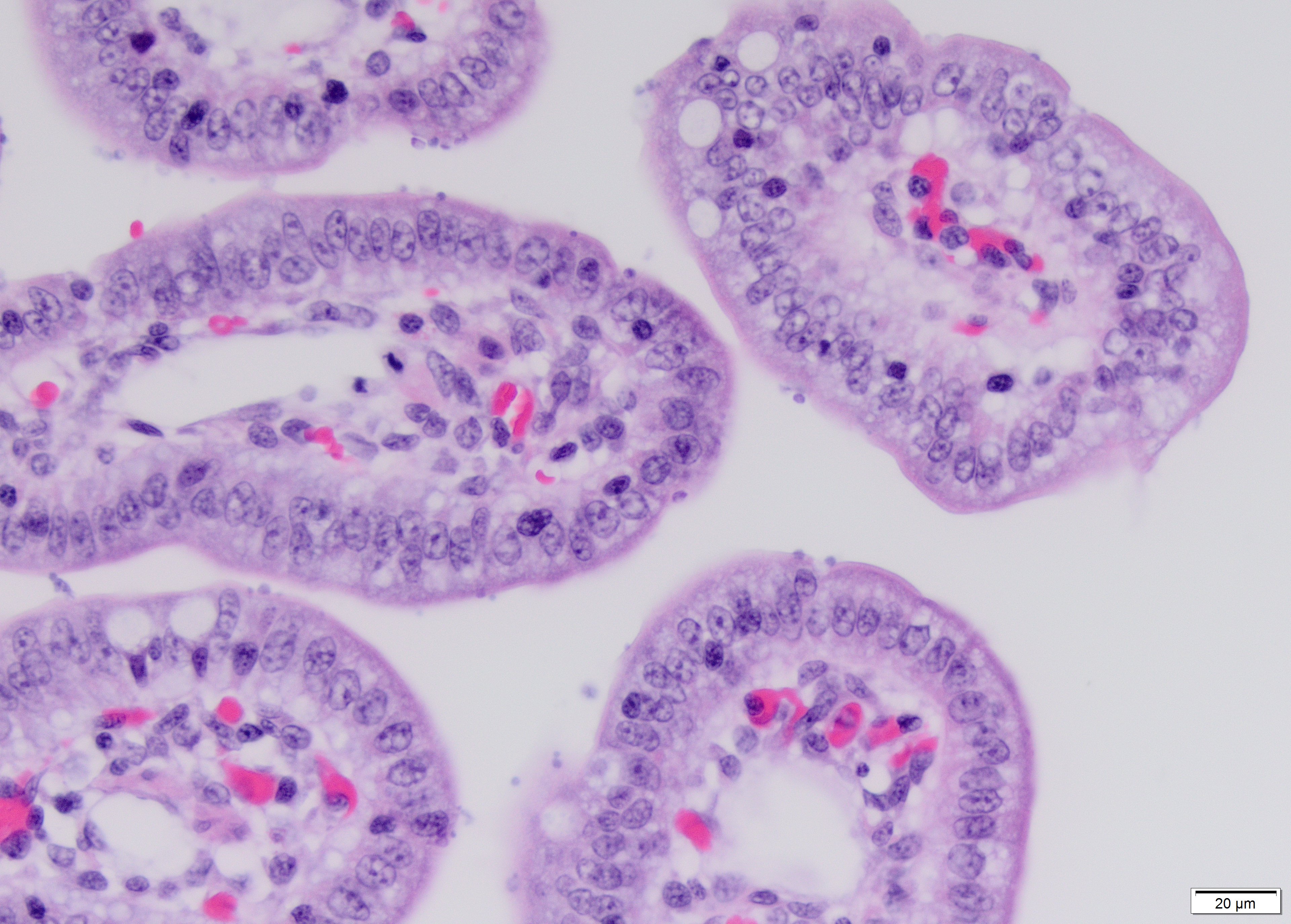

Supplement: S1 Data — (ZIP) [file ppat.1011425.s014.zip › Supporting Information Data/micrographs/Fig. S9/Cassette-vitrified (bottom right).jpg]

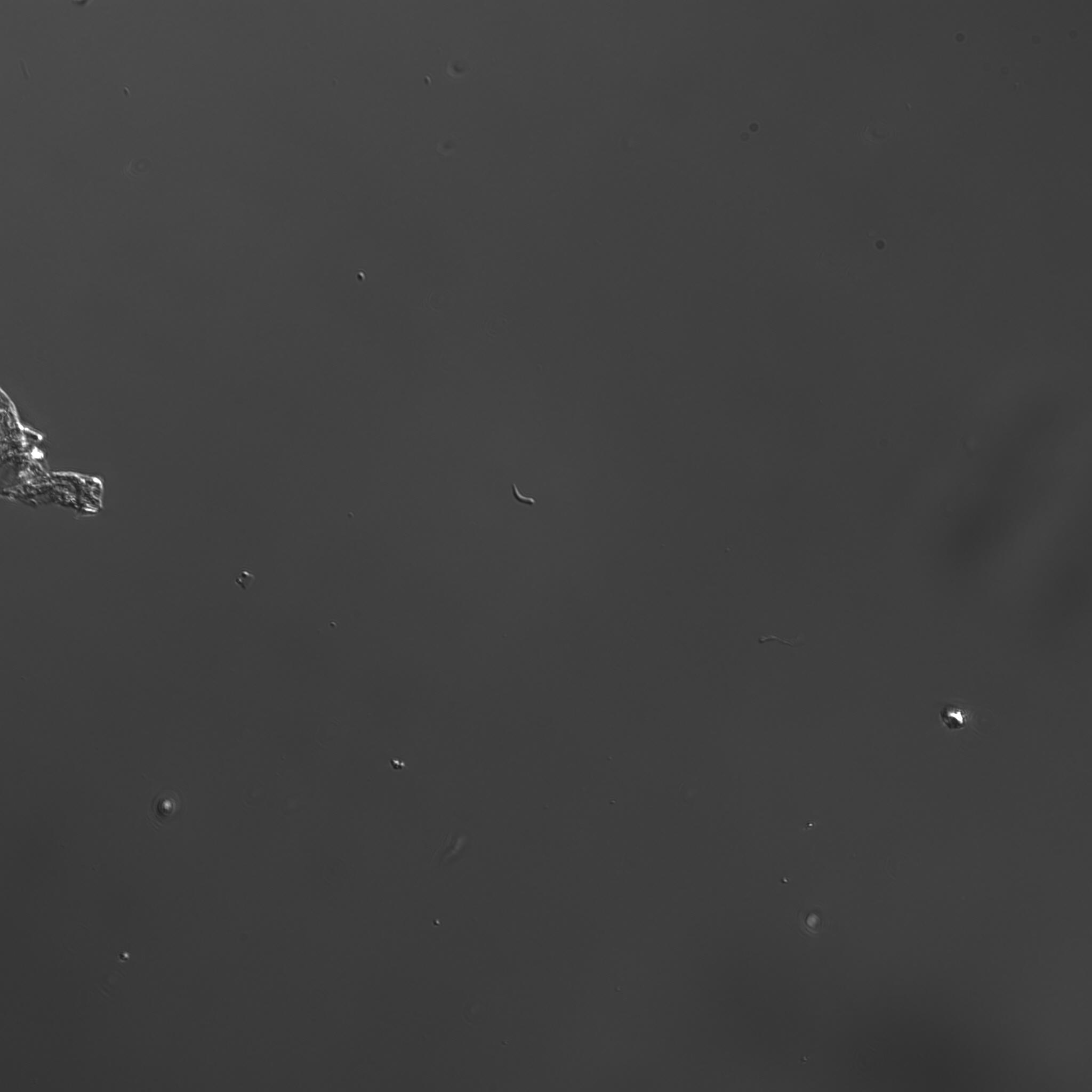

Supplement: S1 Data — (ZIP) [file ppat.1011425.s014.zip › Supporting Information Data/micrographs/Fig. 5b/Positive outcome (top right).jpg]

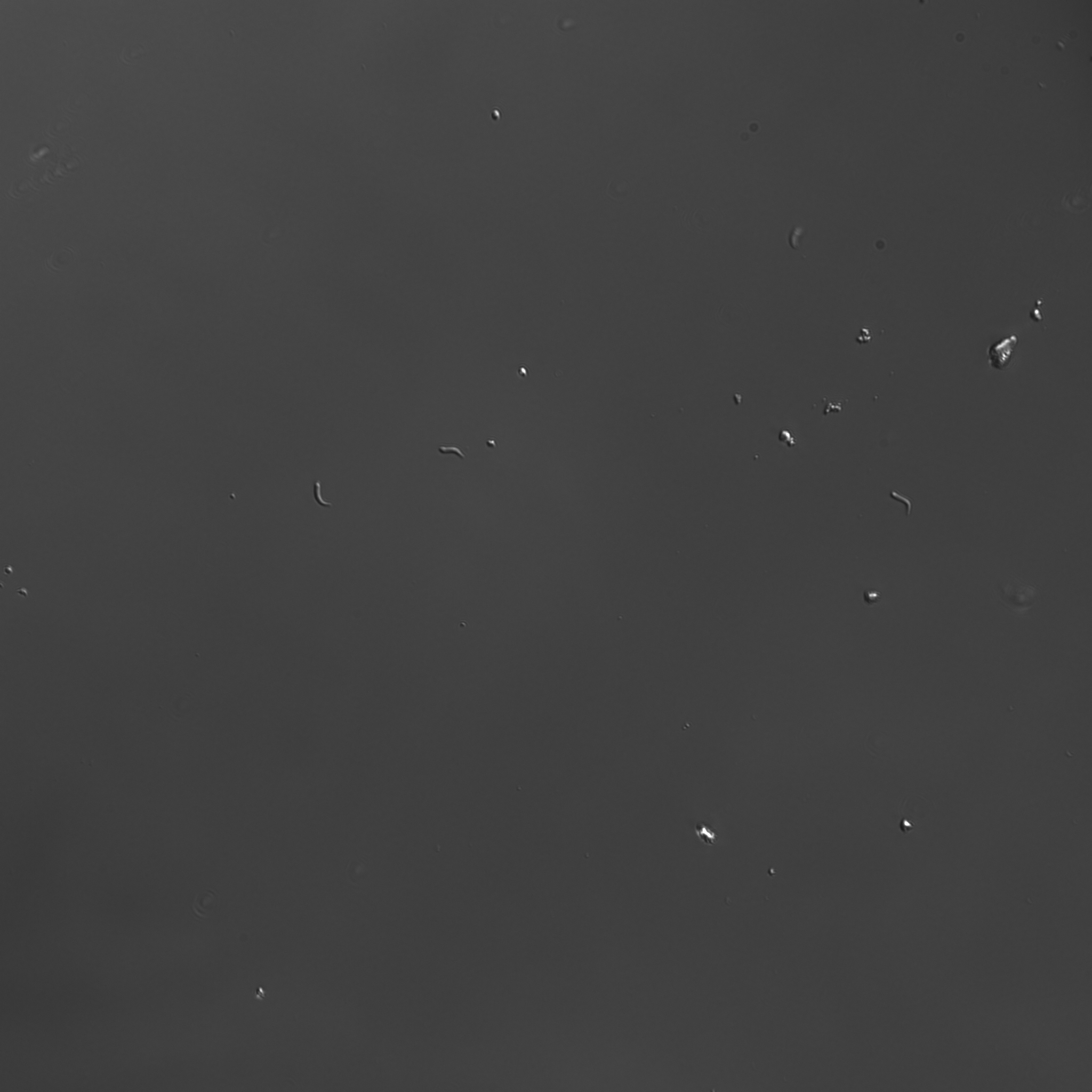

Supplement: S1 Data — (ZIP) [file ppat.1011425.s014.zip › Supporting Information Data/micrographs/Fig. 5b/Positive outcome (top left).jpg]

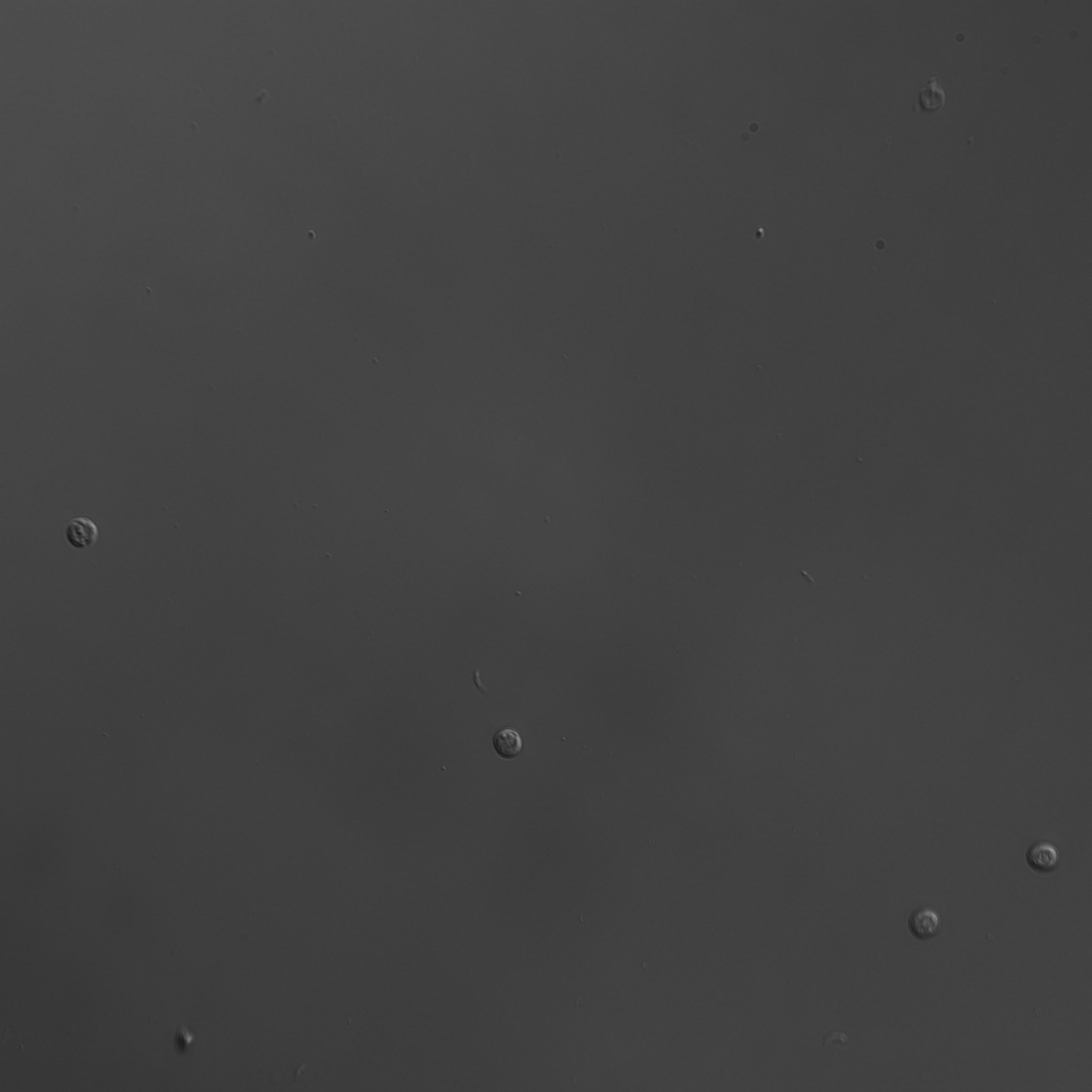

Supplement: S1 Data — (ZIP) [file ppat.1011425.s014.zip › Supporting Information Data/micrographs/Fig. 5b/Negative outcome (bottom left).jpg]

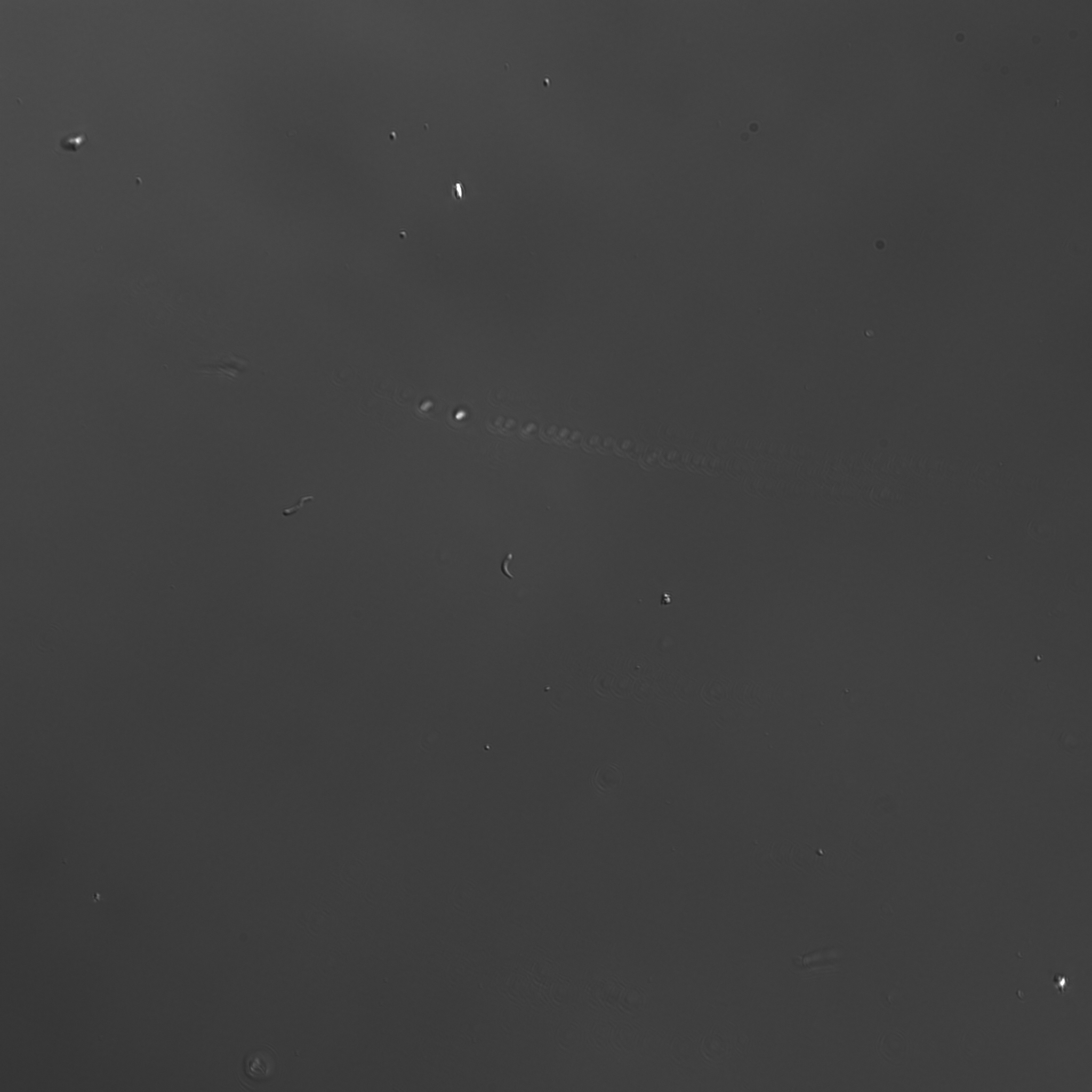

Supplement: S1 Data — (ZIP) [file ppat.1011425.s014.zip › Supporting Information Data/micrographs/Fig. 5b/Positive outcome (top middle).jpg]

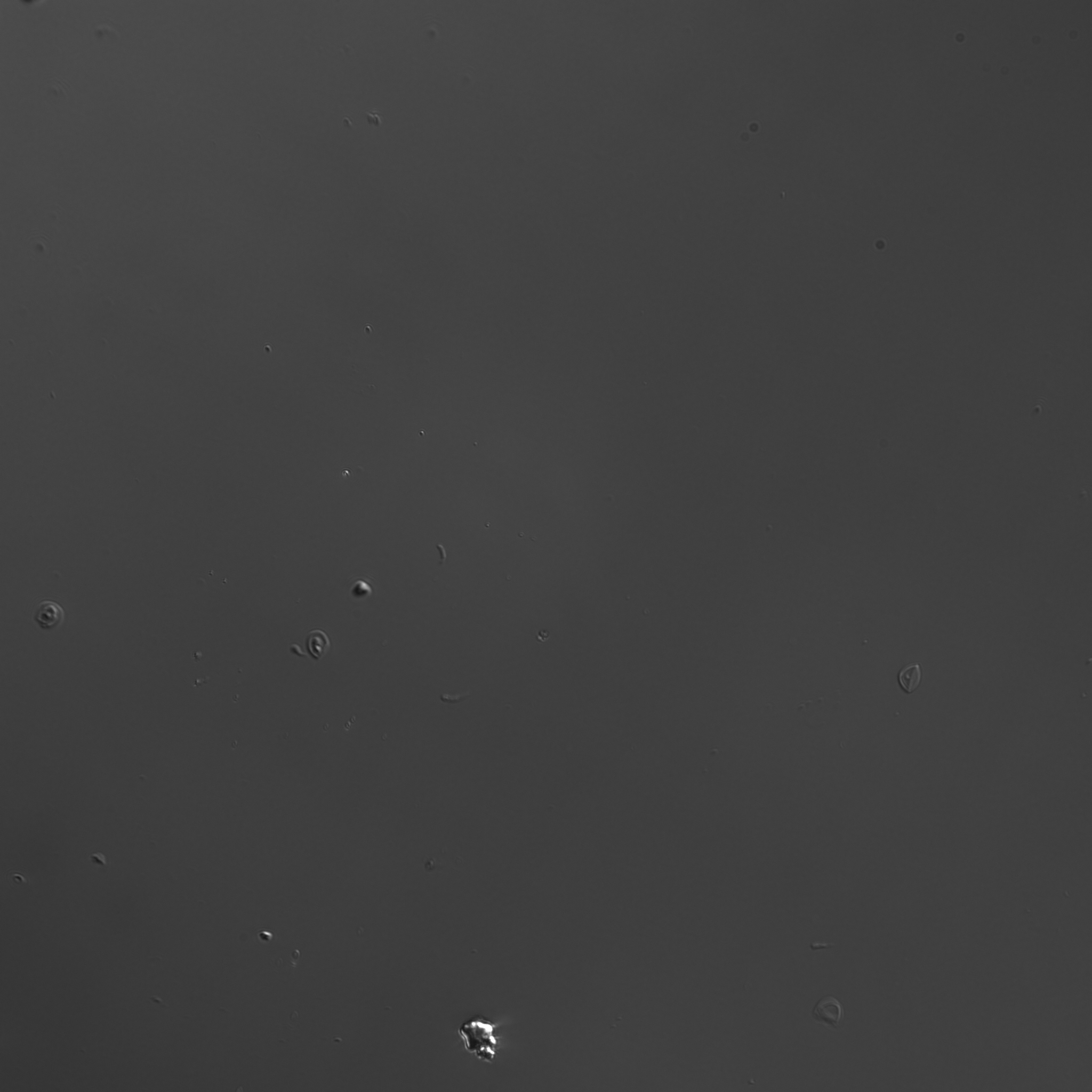

Supplement: S1 Data — (ZIP) [file ppat.1011425.s014.zip › Supporting Information Data/micrographs/Fig. 5b/Negative outcome (bottom middle).jpg]

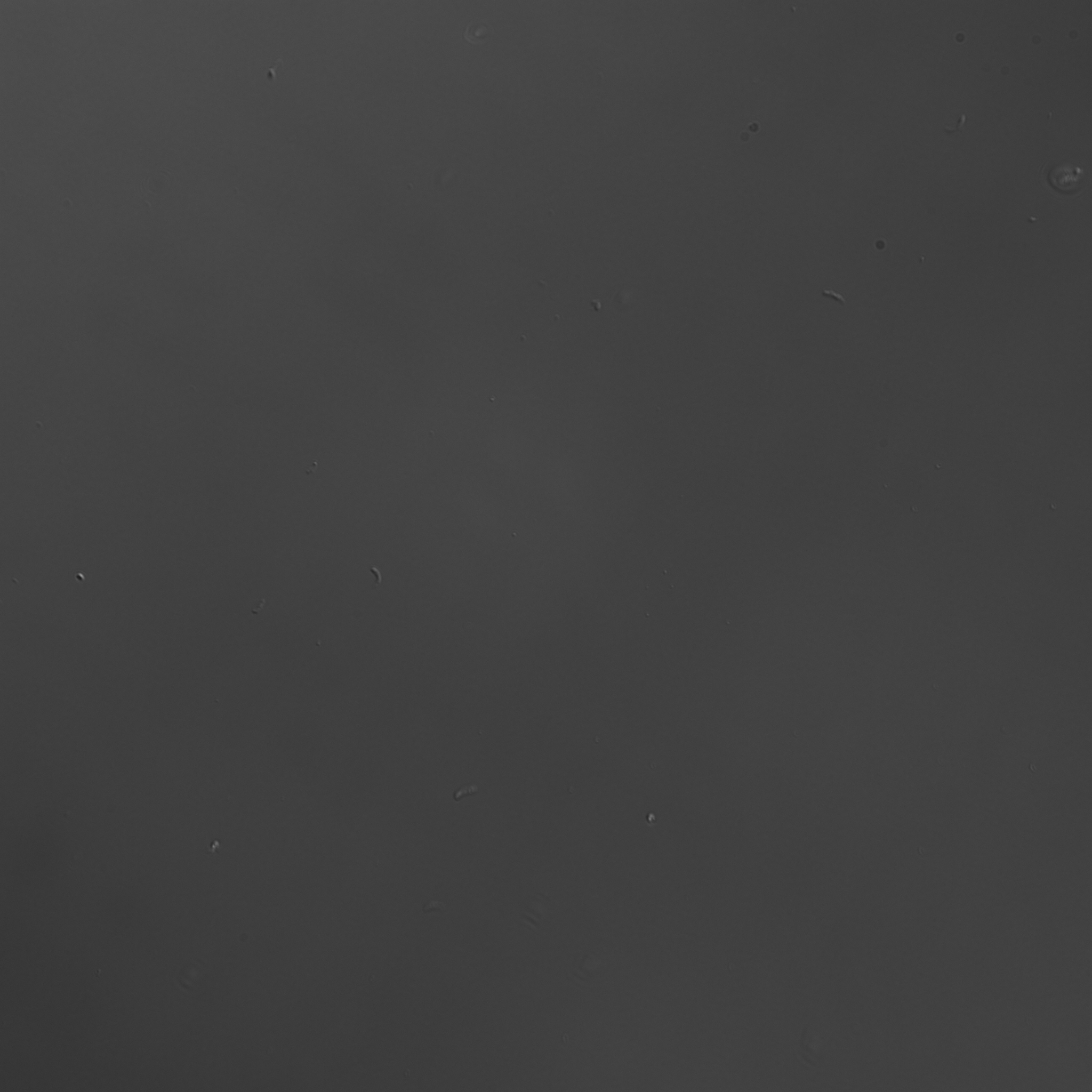

Supplement: S1 Data — (ZIP) [file ppat.1011425.s014.zip › Supporting Information Data/micrographs/Fig. 5b/Negative outcome (bottom right).jpg]

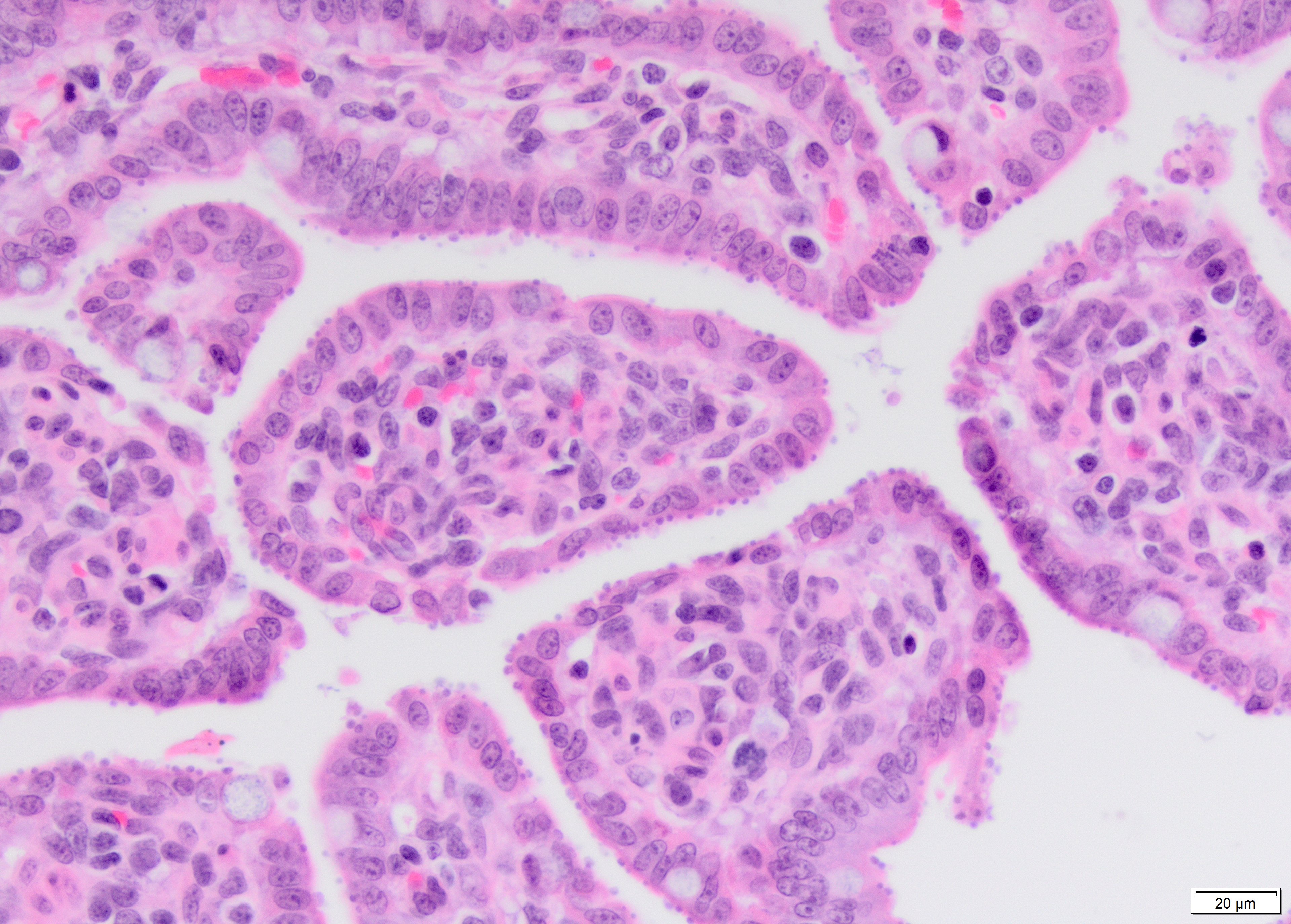

Supplement: S1 Data — (ZIP) [file ppat.1011425.s014.zip › Supporting Information Data/micrographs/Fig. S8/Positive control AF+ (top left).jpg]

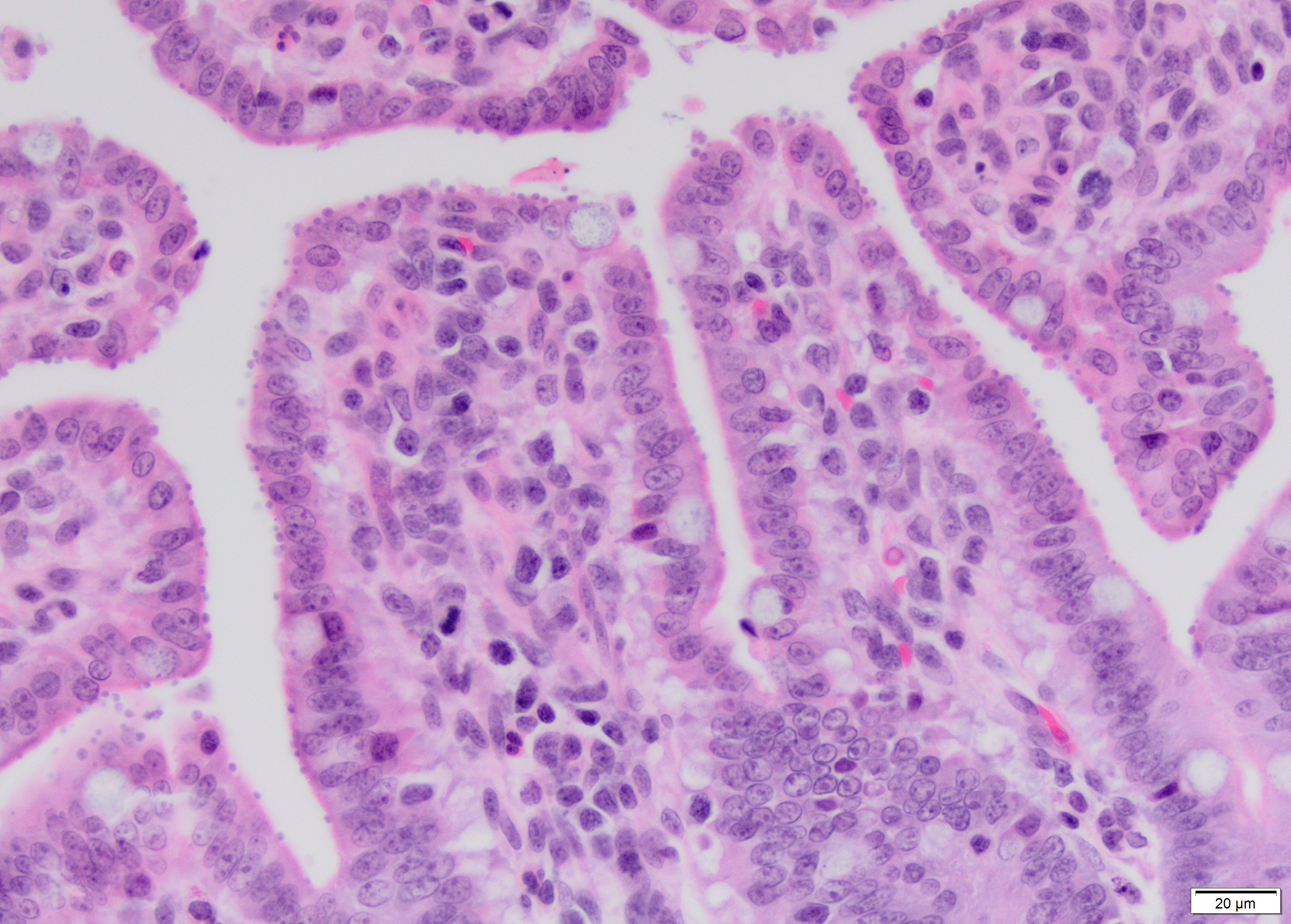

Supplement: S1 Data — (ZIP) [file ppat.1011425.s014.zip › Supporting Information Data/micrographs/Fig. S8/cassette vitrified AF+ (bottm left).jpg]

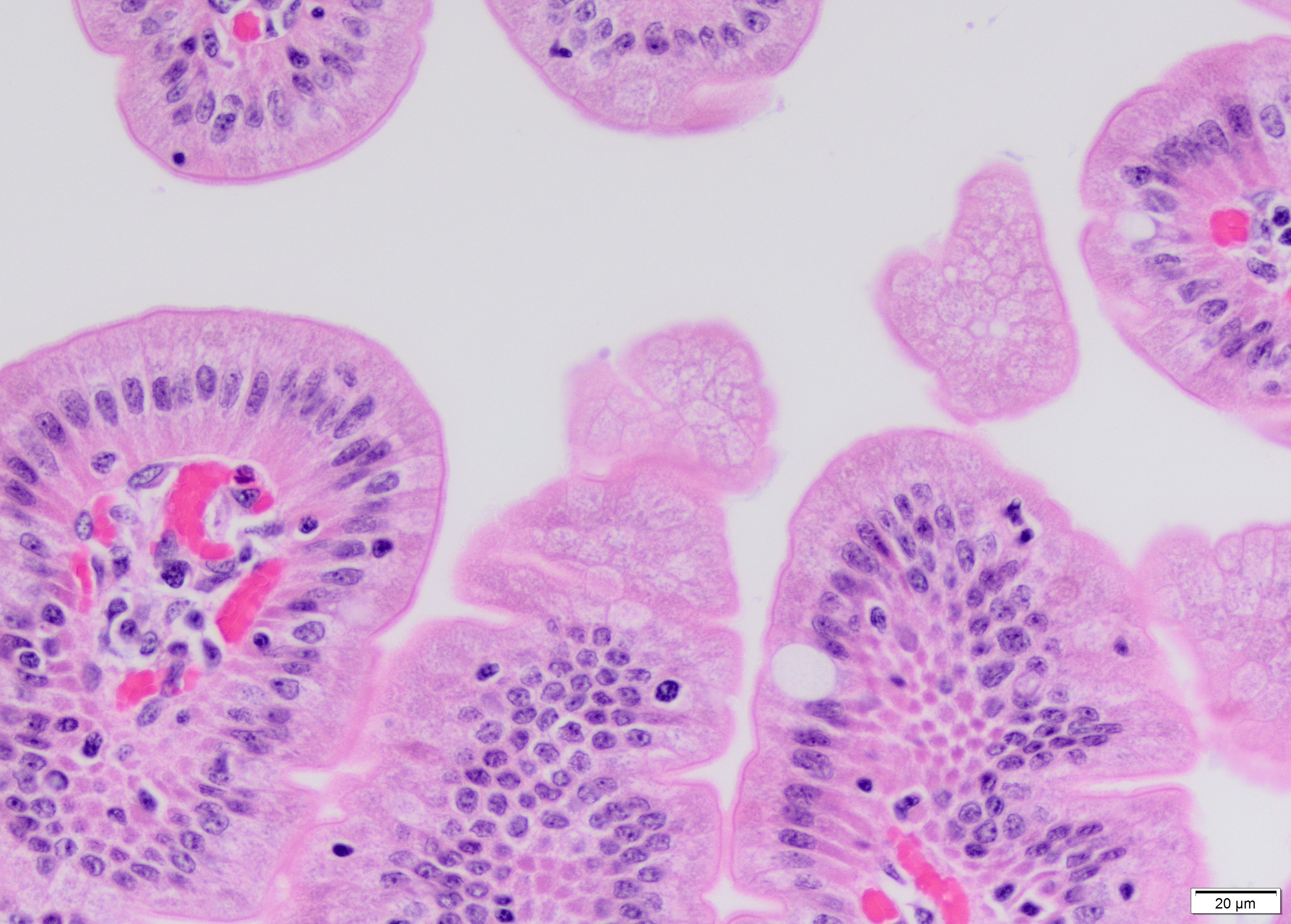

Supplement: S1 Data — (ZIP) [file ppat.1011425.s014.zip › Supporting Information Data/micrographs/Fig. 6b/Negative control (top right).jpg]

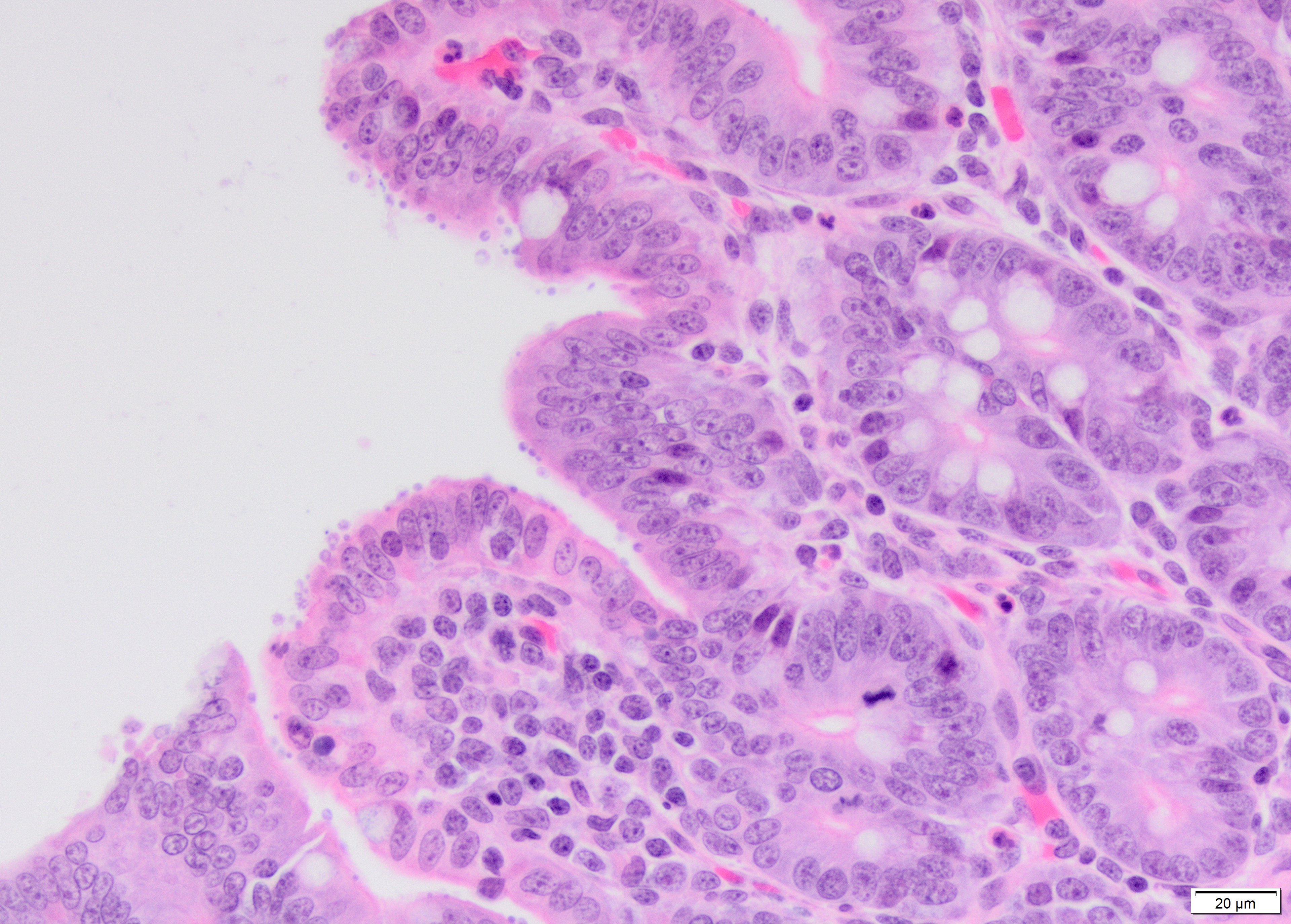

Supplement: S1 Data — (ZIP) [file ppat.1011425.s014.zip › Supporting Information Data/micrographs/Fig. 6b/Cassette-vitrified (AF+)(bottom left).jpg]

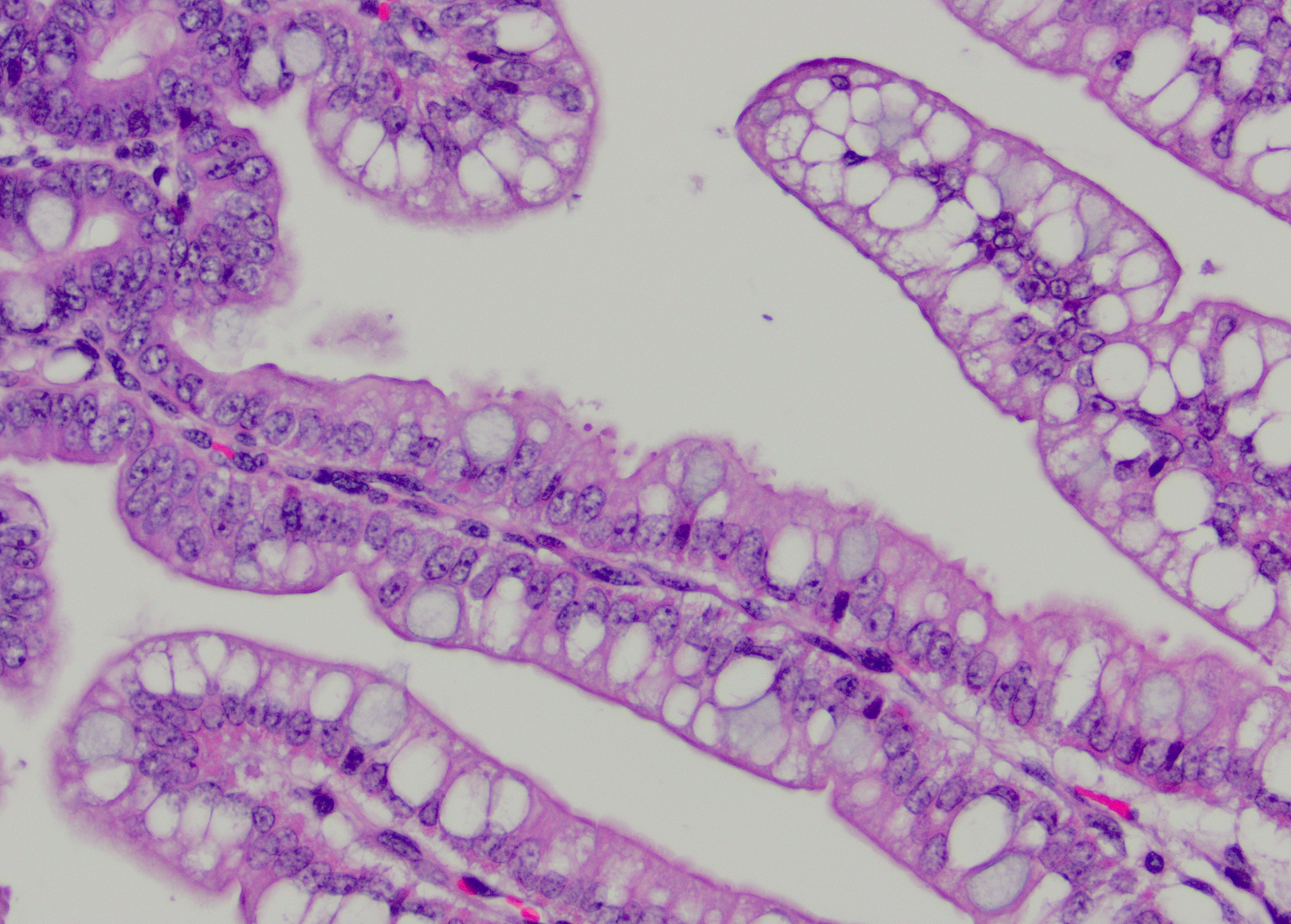

Supplement: S1 Data — (ZIP) [file ppat.1011425.s014.zip › Supporting Information Data/micrographs/Fig. 6b/Cassette-vitrified (AF+)(bottom right).tif]

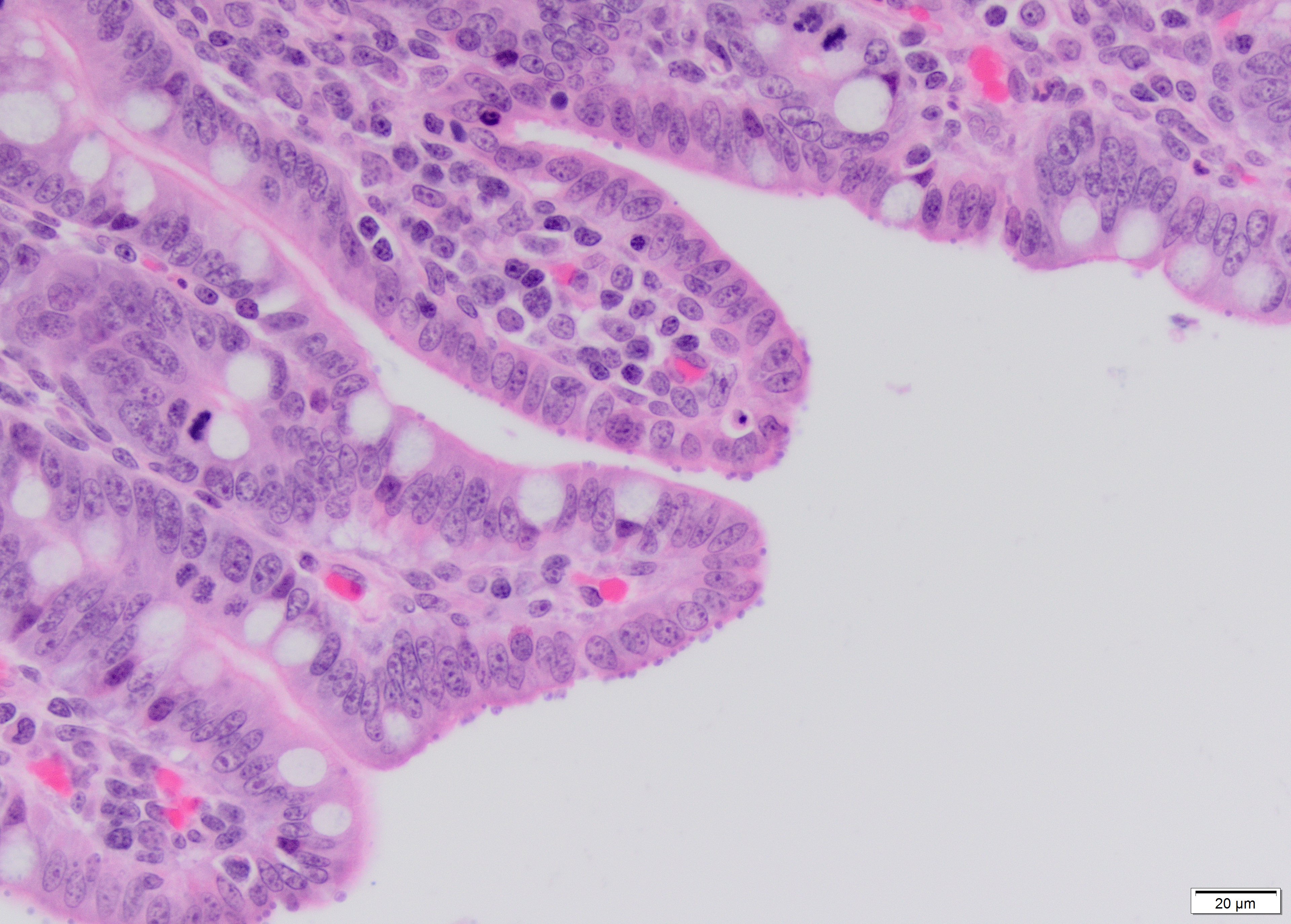

Supplement: S1 Data — (ZIP) [file ppat.1011425.s014.zip › Supporting Information Data/micrographs/Fig. 6b/Fresh control (top left).jpg]
