# Supplementary material for: Scalable cryopreservation of infectious Cryptosporidium hominis oocysts by vitrification
Source: PLoS Pathog. 2023 Jun 8;19(6):e1011425. doi: 10.1371/journal.ppat.1011425 (PMC10284403; doi:10.1371/journal.ppat.1011425)
Supplement: Attachment — Submitted filename: Response to reviewers.docx [file ppat.1011425.s015.docx]

Submission PPATHOGENS-D-23-00245

Title: Scalable cryopreservation of infectious *Cryptosporidium hominis* oocysts by vitrification.

We appreciate the reviewers’ careful analysis of this manuscript and appreciate the points raised. We have addressed these points and revised the manuscript and supplementary information, accordingly. Please see a point-by-point comments of referees (in italics), followed by our response. All changes to the manuscript are highlighted in yellow.

**Editor**

***Comment 1:*** *I might have overlooked this, but as this a pure methods paper, a detailed supplemental step by step protocol that includes where to purchase specialized materials like the cassettes would help to spread the technology throughout the field.*

Response: We agree that detailed protocol and information about materials will be useful for the reader. In our original submission we have included a detailed step-by-step protocol to the Supplementary Information (Supplementary Protocol 1). We now amended this protocol with a list of materials and their sources. The information about cassette availability is also included in Methods section (page 21).

**Reviewer #1**

***Comment 1:*** *Does the same approach work for Cryptosporidium parvum? If the authors have data validating this method with C parvum, it should be included in the supplement, along with any needed protocol modifications. I believe the potential importance of the method is greatest for simpler preservation of the growing number of transgenic C parvum strains than for single-lot production of C hominis that still can't be amplified by recipient labs.*

Response: We have performed three trials comparing the same cryoprotective protocol consisting of thermal permeabilization and vitrification in the cassette between age-matched *C. parvum* Iowa strain and *C. hominis.* Although oocysts survival was assessed only *in vitro* by measurement of viability and quality of excystation, the protocol performs comparably between these two species. Data is now included in Fig. S11 and discussed in Results (lines 267-270, page 12) and Discussion (lines 308-311, page 14) sections. *In vivo* infectivity of *C. parvum* remains to be determined in future studies. We also agree with the reviewer that thermal permeabilization preceding vitrification has a potential to streamline cryopreservation across *Cryptosporidium* species, however this approach needs to be tested amongst species and strains and validated in the animal model of infection.

***Comment 2:*** *I strongly disagree with the word "crucial" in line 330 regarding use of C hominis for clinical challenge trials. "Preferable" would be more accurate. Assuming comparable in vitro susceptibility of C hominis and C parvum, which should be measured for both species (ideally multiple isolates of both species), outcomes in an animal or a person will be dependent on drug exposure at the relevant site and not affected by the species. Studies to determine if the tissue distributions of C hominis and C parvum in people are the same or different may be warranted, but there is currently no reason to believe that outcomes in people would be affected by the species.*

Response: We amended the text as suggested (now in line 384, page 17). We argue however that, while this may be true for drug therapy, immunization with one or the other would likely matter. This is because there is only partial cross-protection between the two species observed in piglets. Additionally challenge studies in human volunteers showed that fewer *C. hominis* oocysts were required to induce a clinical disease in comparison to *C. parvum*.

***Comment 3:*** *The data show clearly that cryopreserved Tu502 C hominis can be recovered by infecting piglets, but with a delay in the onset of shedding, so there is obviously a genetic bottleneck with freezing that may have important implications regarding the utility of the recovered parasites, with perhaps an exaggerated impact for older oocysts. The 16S sequencing in Fig S3 does little to address this. It is possible, for example, that a subset of the Tu502 parent strain has genetic or epigenetic features that enhance survival. This is worthy of mention in the discussion. Future studies to examine differences in the input and recovered populations might be very interesting, such as by whole genome sequencing to identify SNPs correlated with survival. Similarly, it would be very interesting to know if you progressively select for greater survival with sequential rounds of cryopreservation and recovery. Of course, such changes might be correlated with heartiness in other ways. This seems an issue worthy of discussion, as a limitation of the current method and also as a potential route for further investigation.*

Response: The reviewer brings up an important point regarding cryopreservation potentially driving the genetic selection. We did not attempt to test this hypothesis with 18S rRNA sequencing demonstrated in Fig S3, which was performed only to verify oocyst identity after recovery from year-long cryogenic storage and subsequent animal passage. It is unclear whether the observed decreased infectivity of frozen inocula is reflective of the genetic bottleneck or the variability in oocyst permeability, water content, concentration of native biomolecules etc., leading to variable survivability. We agree this is an important consideration and a direction for future investigation that warrants the discussion. Text is now added in lines 347-352, page 16.

***Comment 4:*** *Line 44: Based on the reanalysis of GEMS data, it's no longer accurate to state cryptosporidiosis ranks 2nd amongst pathogens that cause life-threatening diarrhea. This should be corrected, and the GEMS reanalysis should be cited.*

Response: Thank you for reminding us about this important reanalysis study. We have corrected the text indicating that *Cryptosporidium* ranks 4^th^ among children referring to the analysis combining all study sites and age strata and updated the list of citations with Liu et al, 2016 (line 44, page 3).

**Reviewer #2**

***Comment 1:*** *Lines 84-85: I think a longer summary of the method that works for cryopreservation of C. Parvum is required. This includes the fact that you use a mix to trehalose and DMSO, that they are flash frozen in liquid nitrogen in microcapillaries (or the same cassette you use here for large scale freezing of C. Hominis), and stored in liquid phase. I don't think the precendent of this work is stated enough. It is important to demonstrate where the starting point was for C. Parvum and how that was used as a guide for the experiments detailed here. Currently this manuscript feels as if this method was invented de novo with little comparison to the c. Parvum method.*

Response: We have added a clarifying note to the Introduction (lines 87-93, pages 4-5). Per request of Reviewer #3, we have also discussed comparison of methods used for *C. parvum* and *C. hominis* (lines 311-319, pages 14-15).

***Comment 2:*** *Also while it is a huge breakthrough to be able to cryopreserve C. Hominis, I think the authors overlook the challenges of this method. It requires liquid phase liquid nitrogen, access to the cassettes, large number of organisms, and training to be able to carry out this technique. I think it will be helpful to cryopreserve important strains, but will not solve all the current problems suggested in the text. For example, only those who can use the current animal model will be able to invest in this new method. I think a statement about the limitations of this approach should be included.*

Response: We agree with the reviewer there are several limitations to wide implementation of this method, such as a need for technical training, storage in liquid phase of liquid nitrogen and availability of animal models. Access to cassettes is however made possible as they can be purchased from a vendor under catalogue number provided in the manuscript. Please see the discussion about limitations added in lines 360-368, pages 16-17.

***Comment 3:*** *It seems that cryopreservation in microcapillaries will have more board appeal for individual Labotories to implement (doesn't require the cassettes and requires lower numbers of organisms). I think this method should be better highlighted in the manuscript. There will be several labs collecting specimens of C. hominis, and it would be much more feasible for them to cryopreserve their samples in smaller volumes.*

Response: While we agree that in certain situations microcapillary method will be applicable, this method is burdened with many limitations. Most notably, the volume limited to 2 µL per microcapillary requires investment of time and labor involved in freezing and thawing, where each capillary is treated individually and therefore subjects to variability. In contrast, cassette enables freezing and thawing in one event, so that multiple identical inocula are produced from one device. This is particularly beneficial when infecting several piglets, each requiring a dose of ~10^5^. Additional benefits of cassettes are that they can be easily purchased and do not require assembly, are easier and safer to handle, and remain closed during freezing and thawing. However, to better highlight the microcapillary method, we have added an image of the microcapillary to Fig 3 to clarify its use in this set of experiments. In the methods section we also refer the reader to the detailed step-by-step protocol for microcapillary freezing published previously by our group. We have now also added this statement in the Results section (line 181-182, page 9).

***Comment 4:*** *Abstract- the species used to achieve infection rate of 100% should be specified. Is this in vivo? In vitro? Which model? I think it is important to qualify this in the abstract.*

Response: Abstract is amended as suggested.

***Comment 5:*** *Abstract- the abstract does not mention how the method is 'scalable'. I think this should be addressed in the abstract, or if it is not a significant part of the method it should be removed from the title.*

Response: Abstract is corrected to reflect scalability of the method to 100 µL inocula volume.

***Comment 6:*** *Introduction line 64- Location should be added*

Response: Thank you- information is added (now in line 65, page 3).

***Comment 7:*** *line 68 should not be included. This manuscript is about the method of cryopresevation, not about the value of this specific isolate.*

Response: We respectfully disagree with the reviewer. The cryopreservation method described in the manuscript was specifically designed for *C. hominis*, as methods developed previously for *C. parvum* were not compatible. It is the value of this unique isolate that motivated this research.

***Comment 8:*** *Last few sentences of the introduction are too brief. If you wan to summarise the results here, we need more detail (temperature? What is the composition of CPA?, etc).*

Response: We have added general information related to temperature of permeabilization, composition of CPA and validation assays (lines 97-100, page 5). In response to Comment 1, we also included here details on *C. parvum* cryopreservation (lines 86-93, pages 4-5). With these changes, we hope the introduction provides essential information without redundancy.

***Comment 9:*** *Line 90- CPA should be spelled out, rather than abbreviated in the subsection title.*

Response: We changed CPA to cryoprotectant in all subsection and figure titles.

***Comment 10:*** *The only cryoprotective agent (CPA) used here is trehalose. The author should make this clear early on that they are only exploring trehalose. Or they should directly refer to trehalose in the text. By using the phrase CPA, it seems like several agents are being tested, while actually only trehalose is described in the manuscript.*

Response: There are two CPAs used in the protocol: trehalose and DMSO. Trehalose is a non-permeating CPA which remains in extracellular solution, while DMSO permeates intracellularly under permeabilizing conditions. We regret this information was not clearly stated in the original submission. We now clarified across text that the CPA cocktail solution consists of both trehalose and DMSO (line 99, page 5; line 276, page 13, line 299, page 14).

***Comment 11:*** *Line. 131 and line 257- no evidence for this has been presented, so these comments should be removed.*

Response: Regarding sentence in Line 131: “We found that treatment with 1M trehalose followed by a 2 min exposure to 50% DMSO at 37 °C reduces mortality across all ages of oocysts (Fig 2b)…”. We meant it reduces variability in mortality, not mortality itself. Apologies for this omission- the text is now corrected (currently line 143, page 7).

Regarding sentence in Line 257: “Specifically, based on the insignificant cytotoxicity in the presence of high DMSO concentrations, it is inferred that oocyst does not uptake DMSO.” Figures 1b and S1a indicate that *C. hominis* oocysts do not uptake DMSO intracellularly at ambient temperature as there is no toxicity observed after 30 min exposure to 50% DMSO and 30% DMSO after bleaching, respectively. We added reference to these figures accordingly (currently line 279, page 13).

***Comment 12:*** *In detailed protocol, final note detailing overall efficiency of freezing should be moved to the beginning of the protocol. This information is key to understanding how much material can be harvested from freezing, so should be highlighted accordingly.*

Response: Correction was made as suggested. The note now precedes the protocol in the Supplementary Protocol S1.

***Comment 13:*** *Each results section could benefit from having multiple paragraphs to break up ideas.*

Response: We broke down text into paragraphs were appropriate (lines 113, page 6 and 224, page 10).

***Comment 14:*** *How do you store the cassettes in the liquid nitrogen tank? This should be addressed.*

Response: For the purpose of this study cassette storage did not exceed 30 min, which we discuss in lines 353-354, page 16. Here, cassettes were rested at the bottom of a container containing liquid nitrogen. Future effort will focus on devising a secondary container for long-term cryostorage in a liquid nitrogen dewar.

**Reviewer #3**

***Comment 1:*** *Is this newly developed protocol for C. hominis also applicable to cryopreservation of C. parvum oocysts?*

Response: We have performed three trials comparing the same cryoprotective protocol consisting of thermal permeabilization and vitrification in the cassette between age-matched *C. parvum* Iowa strain and *C. hominis.* Although oocysts survival was assessed only in vitro by measurement of viability and quality of excystation, the protocol performs comparably between these two species. Data is now included in Fig. S11 and discussed in Results (lines 267-270, page 12) and Discussion (lines 308-311, page 14) sections. *In vivo* infectivity of *C. parvum* remains to be determined in future studies.

***Comment 2:*** *Could the authors give some discussion to compare this C. hominis protocol and the previous C. parvum protocol (some parameters and procedures)?*

Response: We have added a discussion comparing protocols used for cassette vitrification of *C. parvum* and *C. hominis* (lines 311-319, pages 14-15). We have also clarified that protocol for microcapillary vitrification of *C. parvum* and *C. hominis* remains the same (line 277, page 13).

***Comment 3:*** *Lines 130 – 131 (bile salt): Please specify which one of the bile salts was used. The figure also lacks info on the bile salt.*

Response: The bile salt used was taurocholic acid, as indicated in the caption of Fig. 2c as well as in Methods section (lines 516, page 23 and 526, page 24). We have now also added this information in line 147, page 7. The reviewer correctly notices that Fig. 2c does not mention use of bile salts. This was due to adoption of the nomenclature “induced and spontaneous excystation”, which is explained in the figure caption. For clarity we have now added this information to the legend.

***Comment 4:*** *Lines 225 – 228 (oocysts inoculated to piglets): Please briefly specify the number and batch info for convenience to the reader. Some other places in the Results section might also include brief M&M info, so readers do not have to frequently switch between Results and M&M sections.*

Response: This information is now included across results, figure captions and methods section for all animal experiments to ease interpretation of data (line 180, page 9; line 244, page 11; line 259, page 12, captions to Figs. 6a, S8, S9, S11).

***Comment 5:*** *Lines 228 – 236 (Negative in fecal examination but positive in histology of ileal sections): The investigators monitored oocyst shedding by microscopic examination of fecal smears (acid-fast stained), which might be less sensitive for low number of oocysts. PCR or qPCR might be more sensitive (not a requirement for new experiment, but worth trying if fecal samples are still available).*

Response: Indeed, we reexamined fecal samples from several piglets by qPCR and were able to confirm infection in histology-positive but acid fast-negative piglets. Specifically, this confirmation was made for the experiment summarized in Fig. S8. We did not however perform this analysis for previous experiments and therefore did not report the data.

***Comment 6:*** *References: Most species names are not in italic fonts.*

Response: References are now properly formatted. Thank you.

***Comment 7:*** *Fig. 4: Add unit (mm) to the diagram. Also specify that there are two sets of loading and exit ports (?). In panel (c), specify DMSO concentrations associated with the three forms (ice, ice/glass and glass). These figure annotation suggestions are mostly for easy reading and understanding, and applicable to some other figures.*

Response: We appreciate these suggestions. Fig 4 was modified accordingly. In the figure caption we also clarified that a pair of ports sealed with silicone tabs act as a loading port and a pressure release port duo. We explain the loading and unloading procedures in detail in Methods section (lines 449-457, pages 20-21) and demonstrate it in Fig. S5. Additionally, we updated Fig. S5a to reflect the difference between these two ports.
